# Cross-Cutting mHealth Behavior Change Techniques to Support Treatment Adherence and Self-Management of Complex Medical Conditions: Systematic Review

**DOI:** 10.2196/49024

**Published:** 2024-05-01

**Authors:** Cyd K Eaton, Emma McWilliams, Dana Yablon, Irem Kesim, Renee Ge, Karissa Mirus, Takeera Sconiers, Alfred Donkoh, Melanie Lawrence, Cynthia George, Mary Leigh Morrison, Emily Muther, Gabriela R Oates, Meghana Sathe, Gregory S Sawicki, Carolyn Snell, Kristin Riekert

**Affiliations:** 1 Division of General Pediatrics, Department of Pediatrics, Johns Hopkins School of Medicine, Baltimore, MD, United States; 2 Boston Children’s Hospital, Harvard Medical School, Boston, MA, United States; 3 Division of Pulmonary and Critical Care Medicine, Department of Medicine, Johns Hopkins School of Medicine, Baltimore, MD, United States; 4 Success with Therapies Research Consortium CF Community Member Advisory Board, Bethesda, MD, United States; 5 Cystic Fibrosis Foundation, Bethesda, MD, United States; 6 Children’s Hospital Colorado, University of Colorado School of Medicine, Aurora, CO, United States; 7 Division of Pediatric Pulmonary & Sleep Medicine, Preventive Medicine, University of Alabama at Birmingham, Birmingham, AL, United States; 8 Children’s Health Dallas, University of Texas Southwestern Medical Center, Dallas, TX, United States

**Keywords:** cystic fibrosis, mobile health, technology, self-management, patient adherence, behavior intervention, mHealth intervention, systematic review, evaluation of mHealth, treatment adherence, mHealth

## Abstract

**Background:**

Mobile health (mHealth) interventions have immense potential to support disease self-management for people with complex medical conditions following treatment regimens that involve taking medicine and other self-management activities. However, there is no consensus on what discrete behavior change techniques (BCTs) should be used in an effective adherence and self-management–promoting mHealth solution for any chronic illness. Reviewing the extant literature to identify effective, cross-cutting BCTs in mHealth interventions for adherence and self-management promotion could help accelerate the development, evaluation, and dissemination of behavior change interventions with potential generalizability across complex medical conditions.

**Objective:**

This study aimed to identify cross-cutting, mHealth-based BCTs to incorporate into effective mHealth adherence and self-management interventions for people with complex medical conditions, by systematically reviewing the literature across chronic medical conditions with similar adherence and self-management demands.

**Methods:**

A registered systematic review was conducted to identify published evaluations of mHealth adherence and self-management interventions for chronic medical conditions with complex adherence and self-management demands. The methodological characteristics and BCTs in each study were extracted using a standard data collection form.

**Results:**

A total of 122 studies were reviewed; the majority involved people with type 2 diabetes (28/122, 23%), asthma (27/122, 22%), and type 1 diabetes (19/122, 16%). mHealth interventions rated as having a positive outcome on adherence and self-management used more BCTs (mean 4.95, SD 2.56) than interventions with no impact on outcomes (mean 3.57, SD 1.95) or those that used >1 outcome measure or analytic approach (mean 3.90, SD 1.93; *P*=.02). The following BCTs were associated with positive outcomes: self-monitoring outcomes of behavior (39/59, 66%), feedback on outcomes of behavior (34/59, 58%), self-monitoring of behavior (34/59, 58%), feedback on behavior (29/59, 49%), credible source (24/59, 41%), and goal setting (behavior; 14/59, 24%). In adult-only samples, prompts and cues were associated with positive outcomes (34/45, 76%). In adolescent and young adult samples, information about health consequences (1/4, 25%), problem-solving (1/4, 25%), and material reward (behavior; 2/4, 50%) were associated with positive outcomes. In interventions explicitly targeting medicine taking, prompts and cues (25/33, 76%) and credible source (13/33, 39%) were associated with positive outcomes. In interventions focused on self-management and other adherence targets, instruction on how to perform the behavior (8/26, 31%), goal setting (behavior; 8/26, 31%), and action planning (5/26, 19%) were associated with positive outcomes.

**Conclusions:**

To support adherence and self-management in people with complex medical conditions, mHealth tools should purposefully incorporate effective and developmentally appropriate BCTs. A cross-cutting approach to BCT selection could accelerate the development of much-needed mHealth interventions for target populations, although mHealth intervention developers should continue to consider the unique needs of the target population when designing these tools.

## Introduction

Ever-advancing mobile health (mHealth) technologies hold immense potential to deliver behavior change techniques (BCTs) to diverse audiences, including people with complex medical conditions that involve treatment adherence and other self-management activities. mHealth refers to “medical and public health practice supported by mobile devices, such as mobile phones, patient monitoring devices, personal digital assistants, and other wireless devices” [1]. Common examples include sending smartphone notifications as medication reminders or recording in an app when treatments are completed. Prior mHealth reviews have broadly summarized mHealth interventions as “reminders, education, or behavioral” [2], which included a wide range of study outcomes beyond adherence or self-management [3] or limited the outcome to medication taking [4,5]. Therefore, existing reviews have a limited impact on exactly how mHealth can most effectively support adherence and disease self-management or can be adapted and tailored for chronic illnesses with complex regimens beyond simply taking medicine.

The BCT Taxonomy [6] was created to define discrete, cross-cutting techniques (or approaches) to changing behavior to facilitate the design and evaluation of behavior change interventions, as well as the comparison of BCTs across interventions to identify which BCTs are the most efficacious. The BCT Taxonomy is disease agnostic such that BCTs found to effectively improve treatment adherence and self-management in one complex medical condition should, in theory, generalize to other complex medical conditions with similar adherence and self-management demands. Reviewing mHealth interventions of diseases with complex adherence and self-management demands using BCT Taxonomy could accelerate the design of mHealth solutions by identifying “essential elements” of effective mHealth interventions. Unfortunately, there is no consensus on what essential features should be included in an adherence or self-management mHealth solution for any chronic medical condition.

Our group’s interest in cross-cutting BCTs for adherence and self-management stems from our work with the cystic fibrosis (CF) community. CF is a rare, multisystemic medical condition affecting an estimated 162,428 people worldwide [7]. CF self-management is complex and typically involves a combination of daily oral medications, inhaled treatment, high calorie diet, chest physiotherapy, airway clearance, and exercise [8]. Not surprisingly, people with CF have demonstrated high rates of nonadherence across various aspects of the multicomponent treatment regimen, including low medication adherence (48%-68%) [9,10], nonadherence to caloric goals (24%-40%) [11], and low adherence to airway clearance therapy (28%) [12]. Effective behavioral interventions are needed to promote CF self-management and, in turn, support health outcomes and quality of life. However, rare diseases with complex regimens are rarely the target population for technology developers, and for almost a decade, people with CF have expressed interest in an app but noted that existing apps do not provide the necessary functionality to address their CF management needs [13-15]. A recent search of the Google Play Store (Android) and Apple App Store (iOS) for health-related apps found that only 29 (1.3%) out of 2272 apps address a rare disease population [16], including CF, with none having empirical evidence of their efficacy.

Recognizing that there is a dearth of empirical research on mHealth solutions for treatment adherence and self-management of CF and other rare diseases, we aimed to learn from the BCTs used in effective mHealth interventions for other chronic medical conditions with complex treatment adherence and self-management demands. We, therefore, purposefully designed our systematic review to include people with complex diseases and regimens with overlapping characteristics to CF. Our research questions were (1) Which BCTs have been used in mHealth interventions? and (2) Which BCTs have a positive impact on adherence and self-management behaviors? Differences in BCTs in adult-only studies compared to adolescent and young adult studies were examined, as well as interventions explicitly targeting medicine taking compared to studies targeting broader self-management and other areas of treatment adherence. A systematic review was used because heterogeneity in measuring adherence and self-management outcomes across studies precludes a meta-analysis [17-19] (in contrast to a systematic review, a meta-analysis involves statistically summarizing results across reviewed studies using effect sizes [20]). Our overarching goal was to identify the essential, cross-cutting BCTs delivered via mHealth to effectively facilitate long-term adherence and self-management for people with complex medical regimens, thereby accelerating intervention development, evaluation, and dissemination.

## Methods

### Overview

Standardized search strategies, eligibility evaluations, and data extraction procedures were used (detailed below and in Multimedia Appendix 1). This review was registered with the International Prospective Register of Systematic Reviews (PROSPERO CRD42021224407), in accordance with PRISMA (Preferred Reporting Items for Systematic Reviews and Meta-Analysis) guidelines (Checklist 1).

### Ethical Considerations

As this was a systematic review, institutional review board approval was not required.

### Search Strategy

A literature search in the PubMed, Scopus, Embase, CENTRAL, Web of Science, and PsycINFO databases identified potentially relevant articles published from 2015 through 2020, to enhance relevance to current technology. Given our group’s focus on CF, 2 categories of search terms were used: “CF-specific” and “other chronic conditions,” which included conditions identified by the study authors as having similar adherence and self-management characteristics to CF (eg, conditions with complex daily medical regimens and diseases often diagnosed in childhood, thus involving caregivers in self-management tasks).

### Eligibility Criteria

Peer-reviewed, English language articles published between 2015 and 2020 reporting original empirical findings of mHealth interventions for selected medical conditions and targeting adherence and self-management were included. The mHealth interventions must be accessed on a mobile device (smartphones, cell phones, or tablets, including internet browser programs) and used by a person managing a medical condition or their caregiver.

#### Post Hoc Exclusions

After executing the search strategy, 3 post hoc exclusion criteria were added. People with chronic obstructive pulmonary disease or engaging in pulmonary rehabilitation were excluded, as it was decided that the former population was too different from people with CF and the latter included medical conditions. Reminder-only text messaging and exclusively synchronous telephone or web videoconferencing interventions were excluded, as our interest was in automated BCTs beyond simple reminders and interventions requiring real-time human interaction. Investigations conducted in low- to middle-income countries were excluded due to potential technology access limitations (unreliable internet or cellular service) that would likely affect the types of interventions tested.

### Selection Process

Study records were compiled in a database; duplicates were removed based on DOI number or title. Reviewers (CKE, E McWilliams, DY, TS, and Brandi Blackshear) evaluated each study record (title and citation; blinded double review) for eligibility criteria. The reviewers screened studies for final inclusion and data abstraction using a REDCap (Research Electronic Data Capture; Vanderbilt University) [21,22] form developed for this study.

### Data Collection

#### Overview

A reviewer independently abstracted the study data. A second reviewer read the article, reviewed the initial data abstraction, and identified items of disagreement. Discrepancies were discussed and resolved with all team members. Study characteristics were abstracted for each study in the final review (publication year, study location, study design, sample size, medical condition, age group, and theoretically derived intervention). Missing study details were noted.

#### Key Definitions

The adherence and self-management measurement method was abstracted. Reviewers categorized adherence and self-management measurement as (1) objective behavior (eg, electronic medication monitoring), (2) subjective behavior (eg, patient-reported medication adherence), (3) psychosocial outcome (eg, disease knowledge and adherence self-efficacy), (4) objective health outcome (eg, hemoglobin A_1c_ and viral load), or (5) subjective health outcome (eg, patient-reported asthma control level). Health outcomes were included if the authors conceptualized them as adherence and self-management indicators.

mHealth tools (eg, app and text messaging) and targets of intervention (eg, taking a specific medicine, airway clearance therapy, diabetes self-management activities, dietary recommendations, exercise and physical activity, managing disease activity and symptoms, etc) were abstracted. mHealth intervention results were categorized based on authors’ conclusion of the results as follows:

- Positive: intervention was associated with improved adherence and self-management.
- Negative: intervention was associated with worse adherence and self-management.
- No impact: intervention had no effect on adherence and self-management.
- Mixed: intervention had different effects (positive effect, negative effect, or no impact) on adherence and self-management due to multiple outcome measures and analytic approaches.

The abstracter used information in the manuscript and the BCT Taxonomy to assign discrete BCTs to each intervention component.

#### Risk of Bias

The Revised Cochrane Risk-of-Bias tool (RoB 2) [23] for randomized controlled trials (RCTs) and the Risk of Bias in Non-randomized Studies-of Interventions (ROBINS-I) [24] tool for nonrandomized studies (excluding qualitative studies) were used to assess risk of bias, certainty, and quality of evidence among the studies reviewed. Blinded double assessments were conducted by 2 independent reviewers (RG, IK, E McWilliams, DY, and AD). The RoB-2 assessed risk of bias due to the randomization process, deviations from intended interventions, missing outcome data, measurement of outcome, and selection of the reported result. The ROBINS-I assessed risk of bias due to confounding, deviations from intended interventions, missing data, bias in measurement of outcomes, and bias in selection of the reported result. Discrepancies in ratings were identified and resolved. If multiple outcomes were assessed, an average risk score was calculated to derive a single rating.

### Synthesis

Statistical analyses were conducted in Stata 15 software (StataCorp LLC). Abstracted data were summarized using frequencies and percentages. Subgroup analyses examined differences in study characteristics, including BCTs used, age group (adult only [≥18 years and older] vs adolescent and young adult [11-25 years or sample characterized by authors as “adolescents and young adults”]), study design (RCT vs non-RCT), and whether the intervention was theoretically derived. We also conducted an exploratory subgroup analysis to examine which BCTs appeared the most often in interventions explicitly focusing on medicine taking compared to interventions focusing on self-management and other adherence targets. The results highlight BCTs (1) appearing in ≥5% of studies and (2) with a difference of >10% between positive effects versus no impact on adherence and self-management outcomes. This does not mean that rarely used BCTs are ineffective or that 10% is a verified benchmark of clinically meaningful difference. This pragmatic decision supported the interpretation of a large number of BCTs and comparisons. Statistically significant (*P*<.05) differences in the number of BCTs based on the direction of results were tested using 1-way ANOVA. No effect measures, missing summary statistics or data conversions, or meta-regression were used for this systematic review.

## Results

### Screening Process

**Figure 1.**
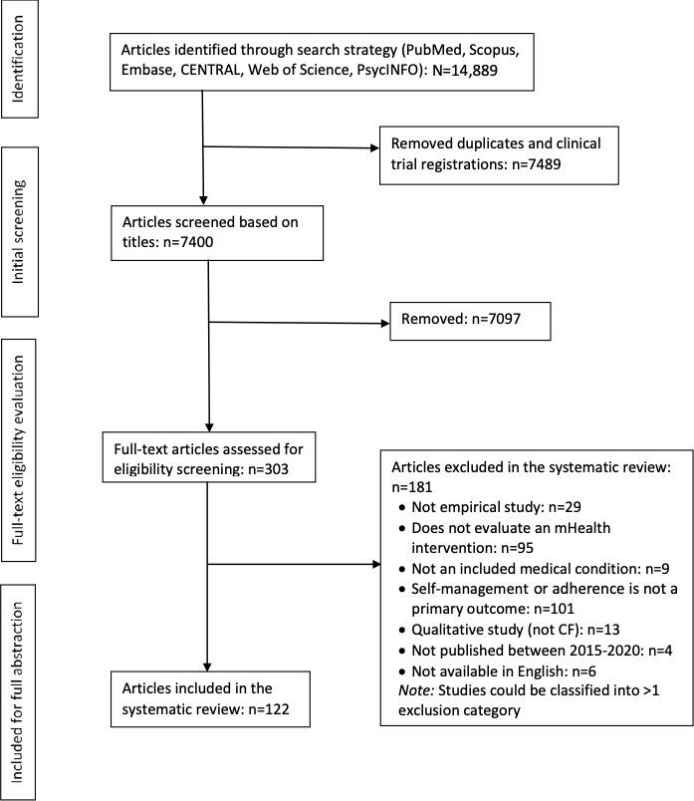
PRISMA (Preferred Reporting Items for Systematic Reviews and Meta-Analysis) diagram. CF: cystic fibrosis; mHealth: mobile health.

Figure 1 presents this review’s PRISMA diagram. The initial search returned 14,889 articles. After removing duplicates and clinical trial registrations, 7400 titles were screened for initial eligibility, 303 articles were potentially eligible, and 122 manuscripts met the criteria for data extraction (see Table S1 in Multimedia Appendix 1 for all included studies and characteristics).

### Study Characteristics

The most represented medical conditions were type 1 or 2 diabetes and asthma (Table 1). Only 6 (4.9%) out of 122 studies involved people with CF. Most studies were published in 2020 (32/122, 26.2%), were conducted outside of the United States (64/122, 52.5%), and used an RCT design (75/122, 61.5%). Nonrandomized study designs were primarily observational pre-post (23/122, 18.9%), observational without pre-post measurement (6/122, 4.9%), or mixed methods (6/122, 4.9%) studies. Study sample sizes ranged from 10 to 14,085 (median 92, IQR 44-179) participants. Most studies involved adult-only (81/122, 66.4%) or adolescent and young adult–only samples (22/122, 18%), followed by child, adolescent, young adult (11/122, 9%); child, adolescent, young adult, and adult (6/122, 4.9%); and child-only (2/122, 1.6%) samples.

**Table 1.** Study characteristics in all studies included for final abstraction (overall) and by direction of primary study results^a^.

Study characteristic		Overall (N=122), n (%)	Positive (n=59), n (%)	No impact (n=23), n (%)	Mixed (n=40), n (%)
**Study design**					
	Randomized controlled trial	75 (61)	26 (44)	21 (91)	28 (70)
	Nonrandomized study	47 (39)	33 (56)	2 (9)	12 (30)
**Use of theory in intervention design**					
	Yes, theory guided intervention design	22 (18)	12 (20)	5 (22)	5 (13)
	Theory mentioned in manuscript, unclear if theory guided intervention design	15 (12)	4 (7)	7 (30)	4 (10)
	No underpinning theory mentioned in manuscript	85 (70)	44 (73)	11 (48)	30 (77)
**Medical condition**					
	Type 2 diabetes	28 (23)	18 (31)	5 (22)	5 (13)
	Asthma	27 (22)	13 (22)	4 (17)	10 (25)
	Type 1 diabetes	19 (16)	7 (12)	4 (17)	8 (20)
	HIV	11 (9)	3 (5)	4 (17)	4 (10)
	Multiple medical conditions	8 (7)	6 (10)	2 (9)	0 (0)
	Inflammatory bowel disease, Crohn disease, or ulcerative colitis	7 (6)	2 (3)	0 (0)	5 (13)
	Cystic fibrosis	6 (5)	3 (5)	1 (4)	2 (5)
	Solid organ transplant	5 (4)	2 (3)	2 (9)	1 (3)
	Diabetes (type not specified)	3 (2)	1 (2)	1 (4)	1 (3)
	Epilepsy	3 (2)	2 (3)	0 (0)	1 (3)
	Kidney disease	2 (2)	0 (0)	0 (0)	2 (5)
	Sickle cell disease	2 (2)	1 (2)	0 (0)	1 (3)
	Rheumatoid arthritis	1 (1)	1 (2)	0 (0)	0 (0)

a Medical conditions are listed from most frequently to least frequently observed in the overall study sample.

Adherence and self-management outcomes were typically evaluated with objective health outcomes or subjective behavior measures (61/122, 50% for each; Table S2 in Multimedia Appendix 1). mHealth interventions were delivered via app (75/122, 61.5%), SMS text messaging (34/122, 27.9%), or website (30/122, 24.6%). Nearly all studies presented mHealth tools used by patients (118/122, 96.7%), but many included health care providers (48/122, 39.3%) or caregivers (21/122, 17.2%). Only 22 (18%) interventions were clearly informed by scientific theory. mHealth interventions most often targeted taking medication (68/122, 55.7%), diabetes self-management activities (46/122, 37.7%), dietary recommendations (32/122, 26.2%), exercise and physical activity (27/122, 22.1%), asthma self-management activities (13/122, 10.7%), managing disease activity and symptoms (11/122, 9%), and general “self-care” behaviors (5/122, 4.1%). One (0.8%) study targeted airway clearance therapy.

Study results were characterized as having a positive effect on the outcome or outcomes (59/122, 48.4%), followed by mixed results (40/122, 32.8%) or no impact (23/122, 18.9%). No studies were characterized as having negative effects (Table 1). Comparing studies reporting positive effects to no impact, 34% (20/59) of the positive studies used objective behavior adherence measures compared to 13% (3/23) of no-impact studies (Table S2 in Multimedia Appendix 1).

### BCTs Used

Across all reviewed studies, 32 different BCTs were used (mean 4.30, SD 2.32). Table S3 in Multimedia Appendix 1 provides the frequencies, definitions, and examples of BCTs appearing in ≥5% of reviewed studies.

### BCTs by Intervention Effect on Outcomes

#### Overview

Interventions with positive effects contained significantly more BCTs (mean 4.95, SD 2.56) than interventions with mixed effects (mean 3.90, SD 1.93) or no impact (mean 3.57, SD 1.95; *P*=.02). BCTs used in >10% of studies with positive results versus no impact (Multimedia Appendix 2) were self-monitoring of behavior, self-monitoring of outcomes of behavior, feedback on outcomes of behavior, feedback on behavior, credible source, and goal setting (behavior).

#### Subgroup Analysis: Age

Table S3 in Multimedia Appendix 1 includes the 15 most common BCTs used in adult-only and adolescent and young adult–only studies. Among adult-only studies (n=81), interventions with positive effects contained significantly more BCTs (mean 5.02, SD 2.13) than studies with mixed effects (mean 4.28, SD 2.27) or no impact (mean 3.28, SD 1.96; *P*=.02). BCTs used in >10% of studies with positive results compared to no impact included prompts and cues, self-monitoring of outcomes of behavior, feedback on outcomes of behavior, self-monitoring of behavior, feedback on behavior, credible source, and goal setting (behavior; Multimedia Appendix 3).

In adolescent and young adult–only studies (n=22), interventions with positive effects contained significantly more BCTs (mean 7.75, SD 5.91) than studies with mixed effects (mean 3.50, SD 1.67) or no impact (mean 5.00, SD 1.41; *P*=.04). BCTs used in >10% of studies with positive results compared to no impact included self-monitoring of behavior, feedback on behavior, goal setting (behavior), information about health consequences, problem-solving, and material reward (behavior; Multimedia Appendix 4).

#### Subgroup Analysis: Study Design and Theory

Non-RCT studies tended to report positive results (33/59, 56%), whereas RCT designs more commonly reported no impact (21/23, 91%) or mixed results (28/40, 70%). Theory rarely guided intervention design; a small proportion (12/59, 20%) of theory-informed interventions were shown to have a positive effect (Table 1).

#### Subgroup Analysis: Intervention Target

Table S4 in Multimedia Appendix 1 includes the 16 most common BCTs used in studies targeting medicine taking versus self-management and other adherence targets (appeared in >5% of studies). A total of 62% (42/68) of studies targeting medicine taking included people with diabetes (21/68, 31%) or asthma (21/68, 31%). A total of 80% (43/54) of studies targeting self-management and other adherence targets included people with diabetes (37/54, 69%) or asthma (6/54, 11%). There were no significant differences in the number of BCTs used in interventions targeting medicine taking (mean 4.07, SD 1.94) compared to interventions targeting self-management and other adherence targets (mean 4.69, SD 2.70; *P*=.16).

Within interventions explicitly targeting medicine taking (n=68), BCTs used in >10% of studies with positive results compared to no impact included prompts and cues, self-monitoring outcomes of behavior, self-monitoring of behavior, feedback on behavior, feedback on outcomes of behavior, and credible source (Multimedia Appendix 5). There were no significant differences in the number of BCTs used based on the direction of results (*P*=.06).

Within interventions focused on self-management and other adherence targets (n=54), BCTs used in >10% of studies with positive results compared to no impact included self-monitoring outcomes of behavior, feedback on outcomes of behavior, self-monitoring of behavior, feedback on behavior, instruction on how to perform the behavior, goal setting (behavior), and action planning (Multimedia Appendix 6). There were no significant differences in the number of BCTs used based on the direction of results (*P*=.21).

### Risk of Bias

Table S1 in Multimedia Appendix 1 reports each study’s risk of bias rating. No study was excluded due to bias rating. For RCTs, 57% (43/75) received an overall risk of bias rating of “Some concerns,” 39% (29/75) had “High” concerns, and only 4% (3/75) had “Low” concerns. “High” concern ratings were generally due to deviations from the intended interventions (18/29, 62%), the randomization process (11/29, 38%), or missing outcome data (11/29, 38%). For nonrandomized studies, 82% (36/44) received an overall risk of bias rating of “Serious” concerns, 9% (4/44) had “Critical” concerns, and 2% (1/44) had “Moderate” concerns. No nonrandomized study had “Low” risk of bias. “Serious” or “Critical” ratings were generally due to confounding (38/40, 95%) or deviations from intended interventions (13/40, 33%).

## Discussion

### Principal Findings

Our literature review of mHealth adherence and self-management interventions returned 122 studies, from which we identified discrete behavioral strategies using the BCT Taxonomy [6] with promise to promote adherence and self-management for people living with medical conditions requiring complex, daily self-management activities. The BCT Taxonomy provides, to date, the most rigorously tested, standardized method to identify cross-cutting BCTs with potential applicability across chronic medical conditions with overlapping adherence and self-management demands. The BCT Taxonomy also helps compare mHealth interventions and provides a shared language about BCTs for clinicians, researchers, mHealth innovators, and other key stakeholders such as patients and caregivers. As technological advances can quickly outdate mHealth, focusing on BCT principles, rather than the technology to deliver them, enhances the research’s relevance and potential generalizability to a range of complex medical conditions, including rare diseases (an area of focus for our group), which often have significant need for such tools in contrast to the finite resources available to conduct large-scale, multistep mHealth design and evaluation studies.

Consistent with prior research [25,26], using more BCTs was associated with improved adherence and self-management. However, 6 BCTs appear particularly promising: self-monitoring of behavior, self-monitoring of outcomes of behavior, feedback on behavior, feedback on outcomes of behavior, credible source, and goal setting. *Self-monitoring of behavior* and *outcomes of behavior* involve tracking health behavior engagement (eg, logging in an app when medicine is taken) or outcomes of behavior (eg, using a Bluetooth-enabled glucometer to monitor blood glucose levels), whereas *feedback on behavior* and *outcomes of behavior* involve providing users with a summarized interpretation of the tracked data (eg, providing in-app graphical representations of one’s daily step count over the past month). Consistent with our results, a prior meta-analysis showed that monitoring medication adherence and providing feedback improve medication adherence [27]. These strategies may build awareness for when the mHealth user engages in a health behavior, provide opportunity to reflect on successes and challenges, and ultimately help the user make informed behavior changes. *Credible source* involves providing expert-generated information about managing the user’s medical condition (eg, the app contains information about etiology, symptoms, and treatment), which presents users with knowledge to understand the condition and its management. *Goal setting (behavior*) involves setting measurable and attainable goals for a target health behavior (eg, set a goal for number of days to exercise in a month), which can help the mHealth user focus on key health behavior and build self-efficacy as goals are met.

Developmental differences emerged between adult samples and adolescents and young adult samples. In adult-only studies, *prompts and cues* (reminders) were associated with positive outcomes, consistent with reviews showing that reminders are associated with a 2- to 3-fold increase in adherence [28,29], but they were less effective in adolescent and young adult studies. Indeed, a pre-post study of children and adolescents with CF found that adherence did not change after delivering reminders only (therefore excluded from this review) for 6 months [30]. Adolescents and young adults may benefit from improving knowledge (*information about health consequences*), improving skills (*problem-solving*), and building motivation (*material reward* [*behavior*]). Given the small number of adolescent and young adult studies, these results and interpretations should be seen as hypothesis generating.

Differences emerged between interventions explicitly targeting medicine taking versus those focused on disease self-management and other adherence targets. In interventions targeting medicine taking, *prompts and cues* and *credible source* were associated with positive outcomes. Reminders and expert information may be the most effective when focused on discrete, clearly defined behaviors rather than complex, multicomponent self-management activities. In interventions focused on self-management and other adherence targets, *instruction on how to perform the behavior, goal setting (behavior),* and *action planning* were associated with positive outcomes. Over three-quarters (43/54, 80%) of studies focused on self-management and other adherence targets involved people with diabetes or asthma, which are relatively common yet complex medical conditions involving self-management behaviors that extend beyond simply taking medicine. Skills training, behavioral goals, and assistance with creating a detailed plan for managing a complex medical condition may be the most effective for multicomponent self-management activities that may involve monitoring and intervening upon changes in disease activity (eg, managing fluctuations in blood glucose levels for people with diabetes or managing asthma exacerbations) and self-managing lifestyle and environmental considerations (eg, diet in diabetes and environmental triggers in asthma). Careful consideration of the intervention target will likely help to further guide appropriate BCT selection from the BCTs found to be associated with improved adherence and self-management in our review.

This review has limitations. A meta-analysis was not conducted due to heterogeneous outcomes [17-19], thus we could not conclude which BCTs were statistically the most effective. Our risk-of-bias assessment highlighted methodological concerns across the studies reviewed. No-impact studies were more likely to be RCTs, and positive studies were more likely to be nonrandomized, raising concerns about publication bias toward positive results irrespective of study quality. We excluded reminder-only interventions; thus, most studies incorporated more than 1 BCT. Our reported average number of BCTs is likely higher than that of all adherence-promoting mHealth interventions. Although we identified some BCTs that may be effective, others may be as or more effective in supporting disease self-management but were rarely used in the reviewed studies. Moreover, no BCT was found to do harm. Thus, mHealth innovators should continue to integrate and evaluate how a wide variety of technology-delivered BCTs may support people living with chronic diseases, including rare diseases such as CF. An inherent limitation of conducting literature reviews is that a cutoff date must be selected, yet scientific literature is constantly being published; there may be utility in conducting an updated systematic review of this topic in the future. We only included studies that were published in peer-reviewed journals to focus on interventions with clear evidence of scientific evaluation; however, expanding our review to “gray literature” may have provided more insight into the most current interventions and reduced publication bias. Our review characterized mHealth BCTs generally. Other metrics including digital literacy and socioeconomic barriers to mHealth were not evaluated. Future researchers should evaluate these factors to support sustained mHealth use among diverse audiences. Additionally, BCTs were analyzed across the included chronic medical conditions given the disproportionate number of studies in diabetes and asthma compared to other medical conditions. Although the BCTs are disease agnostic, intervention developers and researchers should carefully consider the applicability of the BCT to the target patient population.

### Future Directions

Our review identified discrete BCTs that may have broad cross-cutting applicability across chronic diseases with complex medical regimens, including people with CF, the community with which our group primarily works with. We consider our systematic review approach to be a model for gathering key findings from the extant scientific literature to inform the development of multicomponent behavioral mHealth interventions tailored for a patient population that may be smaller and with less existing research, yet has significant self-management needs warranting further research, such as CF [9,10,31,32]. Research involving people with chronic medical conditions following complex treatment regimens should prioritize the design and evaluation of mHealth interventions incorporating cross-cutting, evidence-based, and age-appropriate BCTs to promote adherence and self-management. Such an approach could help accelerate mHealth intervention design and evaluation to create effective products that may be efficiently disseminated to communities with significant need for such tools.

Accelerating mHealth design and evaluation by taking a cross-cutting approach to BCT selection would also help answer remaining “unknowns” about mHealth BCTs and strengthen mHealth intervention quality. For example, although interventions including more BCTs appear to have greater benefit, the optimal number, type, and combination of BCTs to include in mHealth interventions have not been determined. For BCTs demonstrating potential to promote adherence or self-management, the ideal delivery method must be determined (eg, should the BCT *self-monitoring of behavior* be delivered via manual data entry of treatment completion or using an electronic monitoring device to automatically track data?). An overrepresentation of certain BCTs (eg, *prompts and cues* and *self-monitoring of outcomes of behavior*) and underuse of other, potentially more effective techniques (eg, *feedback on behavior* and *goal setting* [*behavior*]) highlight mHealth’s focus on simpler technologies at the expense of innovation and efficacy. Collaborations between behavioral scientists, care teams, patients, caregivers, and industry could answer these questions and produce mHealth solutions that are transformative and effective.

When incorporating BCTs that are expected to effectively and appropriately generalize to a range of complex medical conditions and associated regimens, mHealth intervention developers must still consider the unique needs of the target population. In CF, for example, highly effect CF transmembrane conductance regulator modulator therapies have the potential to simplify the regimen and reduce treatment burden [33,34]. The implementation of key BCTs may need to be adapted as new therapies roll out, although the core theory behind the BCT itself is not expected to change. It is critical to build the scientific evidence base for effective adherence and self-management mHealth interventions that maintain pace with rapidly advancing medical management across complex medical conditions.

## Supplementary material

10.2196/49024 Multimedia Appendix 1 Additional details on search and screening strategies, study characteristics, adherence measure types, behavior change technique definitions, and behavior change techniques by intervention target.

10.2196/49024 Multimedia Appendix 2 The most common BCTs (>5% of all abstracted studies) by the direction of results in the overall sample. BCTs with >10% difference in how often they appear in studies with positive results compared to no-impact results are highlighted in red boxes. BCT: behavior change technique.

10.2196/49024 Multimedia Appendix 3 The most common BCTs (>5% of all abstracted studies) by the direction of results in adult-only samples. BCTs with >10% difference in how often they appear in studies with positive results compared to no-impact results are highlighted in red boxes. BCT: behavior change technique.

10.2196/49024 Multimedia Appendix 4 The most common BCTs (>5% of all abstracted studies) by the direction of results in adolescent and young adult samples. BCTs with >10% difference in how often they appear in studies with positive results compared to no-impact results are highlighted in red boxes. BCT: behavior change technique.

10.2196/49024 Multimedia Appendix 5 The most common BCTs (>5% of all abstracted studies) by the direction of results in interventions explicitly targeting medicine taking. BCTs with >10% difference in how often they appear in studies with positive results compared to no-impact results are highlighted in red boxes. BCT: behavior change technique.

10.2196/49024 Multimedia Appendix 6 The most common BCTs (>5% of all abstracted studies) by the direction of results in interventions targeting self-management or other adherence targets. BCTs with >10% difference in how often they appear in studies with positive results compared to no-impact results are highlighted in red boxes. BCT: behavior change technique.

10.2196/49024 Checklist 1 PRISMA (Preferred Reporting Items for Systematic Reviews and Meta-Analysis) 2020 checklist.

## Abbreviations

BCT: behavior change technique
CF: cystic fibrosis
mHealth: mobile health
PRISMA: Preferred Reporting Items for Systematic Reviews and Meta-Analysis
PROSPERO: International Prospective Register of Systematic Reviews
RCT: randomized controlled trial
REDCap: Research Electronic Data Capture
RoB 2: Revised Cochrane Risk-of-Bias tool
ROBINS-I: Risk of Bias in Non-randomized Studies-of Interventions
