# Supplementary material for: Cross-Cutting mHealth Behavior Change Techniques to Support Treatment Adherence and Self-Management of Complex Medical Conditions: Systematic Review
Source: JMIR Mhealth Uhealth. 2024 May 1;12:e49024. doi: 10.2196/49024 (PMC11085043; doi:10.2196/49024)
Supplement: Multimedia Appendix 1 [file mhealth-v12-e49024-s001.docx]

**Multimedia Appendix 1**

**ADDITIONAL SEARCH AND SCREENING STRATEGIES DETAILS**

**Search Terms**

The “CF-specific” search terms consisted of three categories, 1) medical condition: cystic fibrosis OR CF (CFRD); AND 2) self-management/adherence: self-management OR adherence OR patient compliance OR daily care OR treatment; AND 3) mHealth terms: mobile health (mHealth) OR ehealth OR cell (or) mobile phone OR smartphone OR tablet OR technology OR app OR telehealth OR telemedicine OR telecoaching OR personal digital assistant.

The search terms for “other chronic conditions” consisted of three categories: 1) medical condition: asthma OR COPD OR pulmonary disease OR kidney disease OR dialysis OR transplants OR type 1 diabetes OR type 2 diabetes OR sickle cell disease OR inflammatory bowel disease OR Crohn disease OR ulcerative colitis OR HIV OR epilepsy OR rheumatoid arthritis OR lupus; AND 2) self-management/adherence: as above; AND 3) mHealth terms: as above.

**Level 1 Screening Procedures**

Articles were excluded from review if they met one of the following exclusion criteria: (1) not published in a peer-reviewed journal, (2) not available in English, (3) not published between 2015-2020, (4) not including participants with CF or one of the medical conditions on the inclusion criteria, or (5) clearly a systematic review, meta-analysis, think piece, or published protocol. Reviewers assigned a “1” to include the study or a “0” to exclude a study from the Level 2 screening. Discrepancies in Level 1 screenings were resolved by the lead study author.

**Post-hoc exclusions.** After executing the search strategy and upon further reflection, three post-hoc exclusion criteria were added. First, people with COPD or engaging in pulmonary rehabilitation were excluded from the review as it was decided that these populations were too different from PwCF. Second, reminder-only text messaging and exclusively synchronous telephone or video web-conferencing interventions were excluded, as our interest was in automated BCTs beyond simple reminders and interventions requiring real time human interaction. Investigations conducted in low to middle income countries were excluded due to potential technology access limitations (unreliable internet or cellular service) that would likely affect the types of interventions that could be tested.

**Level 2 Screening Procedures**

One review team member conducted the screening for inclusion, and a second one reviewed the completed screening form. Any disagreements regarding inclusion/exclusion of a study were resolved via discussion by the two reviewers. Inclusion criteria were: an empirical study published between 2015-2020 in English and involving original data collection, evaluating a mobile health intervention as defined for this systematic review, and involving people with CF or another included chronic medical condition; the intervention had to be used by the patient or parent/caregiver; the primary outcome was adherence or self-management as defined by the study authors; if the study was qualitative, the patient sample was people with CF. The lead study author then re-reviewed all screening forms and finalized inclusion decisions. The most common reasons for exclusion at the full text review phase were (1) adherence/self-management was not a primary outcome or (2) the study did not evaluate a mHealth intervention as defined in the current study (see Figure 1).

**Risk of Bias Resolution of Differences**

If overall risk of bias ratings differed by one level (e.g., medium vs. high), the stricter assessment was selected; if the ratings differed by more than one level (e.g., low vs. high), the independent reviewers resolved the discrepancy via group discussion.

| **TABLE S1. STUDY CHARACTERISTICS** | | | | | | | | | | | |
| --- | --- | --- | --- | --- | --- | --- | --- | --- | --- | --- | --- |
|  | **Disease group** | **N** | **Age** | **Study design** | **Intervention’s mobile health technology components** | **User  of intervention** | **Target  of intervention** | **Intervention developed with theory?** | **Behavior  change techniques** | **Effect  on adherence (as reported by study authors)** | **Risk of bias rating** |
| Mary 2019[1] France | Arthritis | 96 | Adult | RCT | Text messaging | Patient | Taking medicine | No | Prompts/cues | Positive effect | High |
| Ahmed 2016[2] Canada | Asthma | 100 | Adult | RCT | Website | Patient, Healthcare provider | Asthma self-management | Yes | Goal setting (outcome) Conserving mental resources Prompts/cues Feedback on outcomes of behavior  Action planning Credible source Self-monitoring of outcomes of behavior  Self-monitoring of behavior Feedback on behavior | Mixed results | Some Concerns |
| Barrett 2017[3] USA | Asthma | 95 | Child, Adolescent and young adult, Adult | Non-randomized experimental design | App, Website, Electronic inhaler | Patient, Caregiver, Healthcare provider | Taking medicine | No | Feedback on behavior Prompts/cues Self-monitoring of behavior  Credible source | Positive effect | Serious |
| Bender 2015[4] USA | Asthma | 899 | Child, Adolescent and young adult | RCT | Phone Calls and Text messaging messages | Caregiver | Taking medicine | No | Prompts/cues Feedback on behavior | Positive effect | Some Concerns |
| Cook 2016[5] UK and the Netherlands | Asthma | 60 | Adult | Non-randomized experimental design | App | Patient | Taking medicine | No | Self-monitoring of behavior Feedback on behavior Feedback on outcomes of behavior  Credible source Prompts/cues Self-monitoring of outcomes of behavior | Positive effect | Serious |
| Davis 2021[6] USA | Asthma | 12 | Adolescent and young adult | Mixed Methods | App | Patient | Taking medicine, Diet, and Exercise/physical therapy | Theory mentioned, unclear if it informed intervention | Goal setting (behavior)  Pros and cons | Mixed results | Serious |
| Khusial 2020[7] UK | Asthma | 42 | Adult | Study 1: RCT  Study 2: Non-randomized experimental design | App, indoor air quality monitor, inhaler adapter, portable spirometer, Fitbit Charge HR | Patient | Taking medicine | No | Self-monitoring of behavior  Adding objects to the environment Feedback on behavior  Self-monitoring of outcomes of behavior Conserving mental resources | Positive effect | Study 1: Low  Study 2: Low |
| Kim 2016[8] South Korea | Asthma | 44 | Adult | RCT | App and Phone Calls | Patient | Asthma self management | No | Self-monitoring of outcomes of behavior Feedback on outcomes of behavior Prompts/cues Action planning | Positive effect | High |
| Kosse 2019[9] the Netherlands | Asthma | 86 | Adolescent and young adult | RCT | App | Patient, Healthcare provider | Taking medicine | No | Self-monitoring of behavior  Self-monitoring of outcomes of behavior Prompts/cues Credible source Feedback on behavior  Social support (unspecified) | Mixed results | Some Concerns |
| Kosse 2019[10] the Netherlands | Asthma | 234 | Adolescent and young adult | RCT | App | Patient, Healthcare provider | Taking medicine, asthma self-management | No | Prompts/cues Self-monitoring of outcomes of behavior  Social support (unspecified)  Self-monitoring of behavior Feedback on behavior Instruction on how to perform the behavior Feedback on outcomes of behavior | Positive effect | High |
| Koufopoulos 2016[11] UK | Asthma | 216 | Adult | RCT | Website | Patient | Taking medicine | Theory mentioned, unclear if it informed intervention | Social support (unspecified)  Self-monitoring of behavior | No effect | High |
| Lin 2020[12] China | Asthma | 21 | Child, Adolescent and young adult | Non-randomized experimental design | App, Propellor inhaler cap | Patient | Taking medicine | No | Feedback on behavior Prompts/cues Problem solving Self-monitoring of behavior Feedback on outcomes of behavior | Positive effect | Critical |
| Ljungberg 2019[13] Sweden | Asthma | 77 | Child, Adolescent and young adult, Adult | RCT | App, Bluetooth spirometer | Patient | Taking medicine | No | Self-monitoring of outcomes of behavior Feedback on outcomes of behavior  Action planning Conserving mental resources | Mixed results | Some Concerns |
| MacDonell 2016[14] USA | Asthma | 49 | Adult | RCT | Text messaging and Website | Patient, Healthcare provider | Taking medicine | Yes | Social support (emotional)  Self-monitoring of behavior Feedback on behavior Prompts/cues Problem solving | Positive effect | Some Concerns |
| Mammen 2022[15] USA | Asthma | 30 | Adult | Non-randomized experimental design | App and Website | Patient, Healthcare provider | Symptom control | No | Self-monitoring of outcomes of behavior Feedback on outcomes of behavior Instruction on how to perform the behavior Conserving mental resources Feedback on behavior | Positive effect | Serious |
| Merchant 2016[16] USA | Asthma | 495 | Child, Adolescent and young adult, Adult | RCT | App, Website, Propellor sensor | Patient, Healthcare provider | Taking medicine | No | Prompts/cues Self-monitoring of behavior  Credible source Feedback on behavior | Mixed results | High |
| Mosnaim 2015[17] USA | Asthma | 12 | Adolescent and young adult | Non-randomized experimental design | App, M-ADEPT electronic monitor | Patient, Healthcare provider | Taking medicine | No | Self-monitoring of behavior Prompts/cues Feedback on behavior  Credible source | Positive effect | Serious |
| Mosnaim 2021[18] USA | Asthma | 100 | Adult | RCT | App and Propellor sensor | Patient | Taking medicine, asthma control | Yes | Self-monitoring of behavior Prompts/cues Social reward Material reward (behavior) Credible source Feedback on behavior | Positive effect | Some Concerns |
| Nemanic 2019[19] Slovenia | Asthma | 100 | Adult | RCT | Text messaging, Website, and Phone Calls | Patient, Healthcare provider | Taking medicine, airway clearance therapy | No | Self-monitoring of outcomes of behavior Prompts/cues Feedback on outcomes of behavior | Mixed results | Some Concerns |
| Newhouse 2016[20] England | Asthma | 148 | Adult | RCT | Website | Patient | Taking medicine, asthma self-management | Theory mentioned, unclear if it informed intervention | Credible source | No effect | High |
| Perry 2017[21] USA | Asthma | 34 | Adolescent and young adult | RCT | App | Patient | Taking medicine | No | Self-monitoring of outcomes of behavior  Credible source Prompts/cues Feedback on outcomes of behavior  Action planning | Mixed results | High |
| Poureslami 2018[22] Canada | Asthma | 106 | Adult | RCT | Text messaging and Website | Patient, Healthcare provider | Asthma control | No | Self-monitoring of outcomes of behavior Prompts/cues Feedback on outcomes of behavior  Action planning | Mixed results | Some Concerns |
| Prabhakaran 2019[23] Singapore | Asthma | 424 | Adult | RCT | Text messaging | Patient, Healthcare provider | Asthma control | No | Self-monitoring of outcomes of behavior Prompts/cues Feedback on outcomes of behavior  Credible source | No effect | High |
| Real 2019[24] USA | Asthma | 40 | Child, Adolescent and young adult | RCT | App | Caregiver | Taking medicine, asthma control | No | Information about health consequences Demonstration of the behavior Feedback on behavior Conserving mental resources  Action planning | Mixed results | High |
| Sleath 2020[25] USA | Asthma | 339 | Adolescent and young adult | RCT | Video and question prompt list on an iPad | Patient, Caregiver, Healthcare provider | Asthma control | Yes | Credible source Reduce prompts/cues | Mixed results | Some Concerns |
| Speck 2016[26] USA | Asthma | 44 | Adult | Mixed Methods | Website | Patient | Taking medicine, asthma control | Yes | Problem solving Credible source Self-monitoring of outcomes of behavior Feedback on outcomes of behavior | Positive effect | Serious |
| Stukus 2018[27] USA | Asthma | 193 | Child | RCT | App | Patient, Caregiver | Taking medicine, asthma control and self-management | No | Prompts/cues Self-monitoring of behavior Feedback on outcomes of behavior  Material incentive (behavior)  Action planning Self-monitoring of outcomes of behavior | No effect | High |
| Vasbinder 2016[28] the Netherlands | Asthma | 209 | Child | RCT | Text messaging, electronic inhaler | Patient, Caregiver | Taking medicine, asthma control | No | Prompts/cues | Positive effect | Low |
| Boon 2020[29] Portugal, Spain, Italy, Belgium, the Netherlands | CF | 171 | Child, Adolescent and young adult | Non-randomized experimental design | App | Patient, Caregiver, Healthcare provider | Taking medicine, Diet | No | Self-monitoring of behavior Information about health consequences Feedback on behavior  Self-monitoring of outcomes of behavior Feedback on outcomes of behavior | Mixed results | Serious |
| Cox 2015[30] Australia | CF | 10 | Adult | Non-randomized experimental design | Website | Patient | Exercise/physical therapy | No | Feedback on behavior  Self-monitoring of behavior Prompts/cues Goal setting (behavior)  Social support (unspecified) | Positive effect | Serious |
| Drabble 2020[31] UK | CF | 14 | Adult | Qualitative | Website, electronic nebulizer | Patient, Healthcare provider | Taking medicine | Yes | Self-monitoring of behavior Feedback on behavior Information about health consequences Demonstration of the behavior  Goal setting (behavior) Prompts/cues Action planning Problem solving | Positive effect | Not evaluated |
| Floch 2020[32] Belgium, Italy, the Netherlands, Portugal, Spain | CF | 154 | Child, Adolescent and young adult, Adult | Mixed Methods | App and Website | Patient, Caregiver, Healthcare provider | Taking medicine, diet | Theory mentioned, unclear if it informed intervention | Goal setting (outcome) Instruction on how to perform the behavior Feedback on behavior  Self-monitoring of behavior  Self-monitoring of outcomes of behavior | Positive effect | Not evaluated |
| Morton 2017[33] UK | CF | 17 | Child, Adolescent and young adult | Non-randomized experimental design | Text messaging | Patient, Caregiver | Taking medicine | No | Prompts/cues | Mixed results | Serious |
| Roehrer 2015[34] Australia | CF | 888 | Child, Adolescent and young adult, Adult | Qualitative | Website | Patient | Symptom monitoring | No | Self-monitoring of outcomes of behavior  Social support (unspecified) Prompts/cues Self-monitoring of behavior Feedback on outcomes of behavior  Self-monitoring of outcomes of behavior Feedback on behavior | No effect | Not evaluated |
| Adu 2020[35] Australia | Diabetes | 50 | Adult | Mixed Methods | App | Patient | Diabetes self-management | Theory mentioned, unclear if it informed intervention | Self-monitoring of behavior Information about health consequences Self-monitoring of outcomes of behavior Feedback on outcomes of behavior  Social support (emotional) Prompts/cues Goal setting (behavior) | Positive effect | Serious |
| Agarwal 2019[36] Canada | Diabetes | 223 | Adult | RCT | App | Patient | Glucose control | Theory mentioned, unclear if it informed intervention | Prompts/cues Monitoring of behavior by others without feedback Self-monitoring of outcomes of behavior  Self-monitoring of behavior | No effect | Low |
| Bajaj 2016[37] Canada | Diabetes | 139 | Adult | RCT | Website | Patient | Taking medicine | No | Self-monitoring of outcomes of behavior Feedback on outcomes of behavior  Action planning | No effect | Some Concerns |
| Boels 2019[38] Germany | Diabetes | 230 | Adult | RCT | App and Text messaging | Patient | Glucose control | Theory mentioned, unclear if it informed intervention | Prompts/cues | No effect | Some Concerns |
| Capozza 2015[39] USA | Diabetes | 93 | Adult | RCT | Text messaging and Website | Patient | Glycemic control | No | Information about health consequences Remove aversive stimulus  Self-monitoring of outcomes of behavior Prompts/cues Feedback on behavior Feedback on outcomes of behavior  Self-monitoring of behavior | No effect | Some Concerns |
| Castensøe-Seidenfaden 2018[40] Denmark | Diabetes | 151 | Adolescent and young adult | RCT | App | Patient, Caregiver, Healthcare provider | Diet, glucose control | No | Social support (unspecified) Credible source Instruction on how to perform the behavior  Social support (practical) Prompts/cues Feedback on behavior | No effect | Some Concerns |
| Chang 2018[41] China | Diabetes | 30 | Adult | Non-randomized experimental design | Private Facebook group | Patient | Taking medicine, Diet, Exercise/physical therapy, foot care, self-monitoring blood glucose |  | Self-monitoring of behavior  Credible source Social support (unspecified) | Mixed results | Serious |
| Chao 2019[42] Taiwan, China | Diabetes | 121 | Adult | RCT | App | Patient, Healthcare provider | Taking medicine, Diet, Exercise/physical therapy, self-monitoring, health coping, risk reduction | No | Self-monitoring of behavior  Credible source Goal setting (behavior) Feedback on behavior  Self-monitoring of outcomes of behavior | Positive effect | Some Concerns |
| Chatzakis 2019[43] Greece | Diabetes | 80 | Child, Adolescent and young adult | RCT | App | Patient | Blood glucose monitoring and control | No | Conserving mental resources  Self-monitoring of outcomes of behavior Demonstration of the behavior | Positive effect | Some Concerns |
| Clements 2017[44] USA | Diabetes | 81 | Adolescent and young adult | Retrospective study | App, glucometer-based data synchronization | Patient, Caregiver, Healthcare provider | Blood glucose monitoring and control | No | Self-monitoring of behavior Feedback on outcomes of behavior  Self-monitoring of outcomes of behavior | Mixed results | Serious |
| Davis 2017[45] USA | Diabetes | 51 | Adult | Non-randomized experimental design | Website | Patient, Healthcare provider | Taking medicine, Diet, and Exercise/physical therapy | Yes | Credible source | Positive effect | Serious |
| Dobson 2015[46] New Zealand | Diabetes | 42 | Adult | Non-randomized experimental design | Text messaging | Patient, Healthcare provider | Taking medicine, Diet, Exercise/physical therapy, diabetes self-management | Yes | Prompts/cues Self-monitoring of outcomes of behavior Feedback on outcomes of behavior  Social support (unspecified) Credible source Goal setting (behavior) | Positive effect | Critical |
| Dobson 2018[47] New Zealand | Diabetes | 366 | Adult | RCT | Text messaging | Patient | Diet, Exercise/physical therapy, and blood glucose monitoring, stress management, foot care | Theory mentioned, unclear if it informed intervention | Prompts/cues Self-monitoring of outcomes of behavior  Credible source Feedback on outcomes of behavior | Positive effect | Some Concerns |
| Garg 2017[48] USA | Diabetes | 100 | Adult | RCT | App and integrated glucometer | Patient, Healthcare provider | Worry and fear of hypoglycemic episode | No | Self-monitoring of outcomes of behavior Feedback on outcomes of behavior Instruction on how to perform the behavior  Goal setting (behavior) | Positive effect | High |
| Gatwood 2016[49] USA | Diabetes | 48 | Adult | RCT | Text messaging | Patient | Taking medicine | Yes | Prompts/cues | No effect | High |
| Gimbel 2020[50] USA | Diabetes | 229 | Adult | RCT | App, Bluetooth-enabled scale, glucometer, blood pressure reader, activity monitor | Patient, Healthcare provider | Taking medicine, Diet, Exercise/physical therapy, blood glucose monitoring weight monitoring, foot care | No | Self-monitoring of outcomes of behavior Feedback on outcomes of behavior Prompts/cues | Mixed results | High |
| Gong 2020[51] Australia | Diabetes | 187 | Adult | RCT | App, Website, optional Bluetooth-enabled glucometer | Patient | Taking medicine, Diet, Exercise/physical therapy, blood glucose monitoring, foot care | Theory mentioned, unclear if it informed intervention | Feedback on outcomes of behavior  Credible source Self-monitoring of outcomes of behavior Instruction on how to perform the behavior  Social support (unspecified) | No effect | Some Concerns |
| Goyal 2017[52] Canada | Diabetes | 92 | Adolescent and young adult | RCT | App, Bluetooth-enabled glucometer | Patient | Taking medicine, Diet, and glucose monitoring | No | Self-monitoring of outcomes of behavior Prompts/cues Material reward (behavior) Feedback on outcomes of behavior  Social support (unspecified) | Mixed results | High |
| Gunawardena 2019[53] Sri Lanka | Diabetes | 67 | Adult | RCT | App | Patient | Taking medicine, Diet, and Exercise/physical therapy | No | Self-monitoring of outcomes of behavior Prompts/cues Conserving mental resources Feedback on outcomes of behavior | Positive effect | High |
| Guo 2015[54] Taiwan | Diabetes | 28 | Adult | Non-randomized experimental design | App | Patient, Healthcare provider | Diabetes self-management | No | Instruction on how to perform the behavior  Self-monitoring of outcomes of behavior Feedback on outcomes of behavior Feedback on behavior  Social support (unspecified) Action planning Prompts/cues Self-monitoring of behavior | Positive effect | Serious |
| Hannon 2018[55] USA | Diabetes | 97 | Adolescent and young adult | RCT | App, Text messaging, integrated glucometer | Patient, Caregiver, Healthcare provider | Diabetes self-care | No | Self-monitoring of outcomes of behavior Prompts/cues Feedback on outcomes of behavior  Action planning | No effect | High |
| Herbert 2015[56] USA | Diabetes | 23 | Adolescent and young adult | Non-randomized experimental design | Text messaging | Patient | Blood glucose monitoring | No | Self-monitoring of outcomes of behavior Prompts/cues | Mixed results | Serious |
| Hilliard 2020[57] USA | Diabetes | 80 | Adult | RCT | App and Text messaging | Caregiver | Supportive family diabetes management | Yes | Instruction on how to perform the behavior  Self-monitoring of behavior Prompts/cues Conserving mental resources Feedback on behavior | No effect | Some Concerns |
| Hooshmandja 2019[58] Iran | Diabetes | 51 | Adult | Non-randomized experimental design | App | Patient | Taking medicine, Diet, Exercise/physical therapy, smoking, foot care | No | Self-monitoring of behavior Feedback on outcomes of behavior Prompts/cues Self-monitoring of outcomes of behavior | Positive effect | Serious |
| Jackson 2021[59] USA | Diabetes | 40 | Adult | Non-randomized experimental design | Text messaging | Patient, Healthcare provider | Taking medicine, Diet, Exercise/physical therapy, blood glucose monitoring | No | Feedback on behavior Prompts/cues Credible source | Positive effect | Serious |
| Jeong 2018[60] South Korea | Diabetes | 338 | Adult | RCT | Smart Care Unit (SCU), a personal tablet allowing for communication between patients and endocrinologists with an auto-transmitter system for glucose concentration | Patient, Healthcare provider | Diet, Exercise/physical therapy, blood glucose monitoring | No | Feedback on behavior  Self-monitoring of behavior  Credible source Self-monitoring of outcomes of behavior Feedback on outcomes of behavior | Mixed results | Some Concerns |
| Kaushal 2020[61] USA | Diabetes | 165 | Adolescent and young adult | RCT | Text messaging |  | Diabetes self-management | No | Prompts/cues Information about health consequences Material reward (behavior) | Mixed results | High |
| Kim 2016[62] South Korea | Diabetes | 29 | Adult | Non-randomized experimental design | App, Bluetooth-enabled glucometer, wearable physical activity monitoring device | Patient | Blood glucose control | No | Self-monitoring of outcomes of behavior Feedback on outcomes of behavior  Action planning Self-monitoring of behavior Conserving mental resources Demonstration of the behavior  Goal setting (behavior) Social support (unspecified) Prompts/cues Feedback on behavior  Credible source | Positive effect | Serious |
| Kim, M 2019[63] USA | Diabetes | 215 | Adult | Non-randomized experimental design | Website, Text messaging | Patient, Healthcare provider | Diet and Exercise/phsical therapy | Yes | Goal setting (behavior) Instruction on how to perform the behavior Prompts/cues Self-monitoring of behavior Feedback on outcomes of behavior  Social support (unspecified) | Positive effect | Serious |
| Kim, E 2019[64] South Korea | Diabetes | 172 | Adult | RCT | App, Bluetooth-enabled glucometer, physical activity tracker | Patient, Healthcare provider | Taking medicine, Diet, and Exercise/physical therapy | No | Feedback on behavior  Self-monitoring of behavior  Goal setting (behavior)  Social support (unspecified) Social support (practical) Prompts/cues Self-monitoring of outcomes of behavior Feedback on outcomes of behavior | Mixed results | Some Concerns |
| Kjos 2019[65] USA | Diabetes | 51 | Adult | Non-randomized experimental design | App | Patient | Taking medicine, Diet, Exercise/physical therapy, blood glucose monitoring | Theory mentioned, unclear if it informed intervention | Restructuring the physical environment Prompts/cues Conserving mental resources Self-monitoring of outcomes of behavior | Mixed results | Serious |
| Klee 2018[66] Switzerland | Diabetes | 55 | Child, Adolescent and young adult | RCT | App | Patient, Caregiver, Healthcare provider | Taking medicine and Diet | No | Feedback on behavior Prompts/cues Conserving mental resources Self-monitoring of outcomes of behavior Feedback on outcomes of behavior Self-monitoring of behavior | Positive effect | High |
| Koot 2019[67] Singapore | Diabetes | 100 | Adult | Non-randomized experimental design | App, integrated scale | Patient, Healthcare provider | Diabetes self-management | No | Credible source Self-monitoring of behavior Feedback on behavior Self-monitoring of outcomes of behavior | Positive effect | Moderate |
| Ku 2020[68] South Korea | Diabetes | 40 | Adult | RCT | App, integrated glucometer | Patient, Healthcare provider | Blood glucose monitoring, diet, exercise | No | Instruction on how to perform the behavior Goal setting (behavior) Self-monitoring of behavior Credible source Feedback on outcomes of behavior Feedback on behavior Conserving mental resources Self-monitoring of outcomes of behavior Social support (practical) | Mixed results | Some Concerns |
| Kumar 2018[69] USA | Diabetes | 146 | Adult | Non-randomized experimental design | App | Patient, Healthcare provider | Taking medicine, Diet, Exercise/physical therapy, blood glucose monitoring | No | Self-monitoring of behavior Self-monitoring of outcomes of behavior Prompts/cues Social support (unspecified) Credible source Feedback on behavior Feedback on outcomes of behavior | Positive effect | Serious |
| Lee 2020[70] South Korea | Diabetes | 66 | Adult | RCT | App | Patient, Healthcare provider | Diabetes self-management | No | Self-monitoring of behavior Self-monitoring of outcomes of behavior Social support (unspecified) Credible source Prompts/cues Feedback on outcomes of behavior | Positive effect | High |
| Lim 2016[71] South Korea | Diabetes | 100 | Adult | RCT | App, Website, and home gateway system (hub) to transfer data from devices to server | Patient, Healthcare provider | Diet, Exercise/physical therapy, blood glucose control | No | Self-monitoring of behavior Prompts/cues Credible source Feedback on outcomes of behavior Self-monitoring of outcomes of behavior Feedback on behavior | Positive effect | Some Concerns |
| Lin 2020[72] USA | Diabetes | 14085 | Adult | Retrospective study | App, integrated glucometer | Patient, Caregiver, Healthcare provider | Diet, Exercise/physical therapy, and blood glucose monitoring | No | Self-monitoring of outcomes of behavior Feedback on outcomes of behavior Prompts/cues Credible source Material reward (behavior) Instruction on how to perform the behavior | Positive effect | Serious |
| McGill 2020[73] USA | Diabetes | 301 | Adolescent and young adult | RCT | Text messaging | Patient | Blood glucose control | No | Prompts/cues Credible source Social reward Problem solving Self-monitoring of outcomes of behavior Social support (emotional) Goal setting (outcome) | Mixed results | High |
| McLeod 2020[74] New Zealand | Diabetes | 429 | Adult | RCT | App and Website | Patient | Blood glucose control | Yes | Goal setting (behavior) Social support (unspecified) Credible source Prompts/cues Self-monitoring of behavior Self-monitoring of outcomes of behavior | No effect | Some Concerns |
| Miller 2017[75] USA | Diabetes | 20 | Adult | Non-randomized experimental design | Text messaging | Patient | Blood glucose control, retinopathy screening rates, and self-care behaviors | No | Prompts/cues | Positive effect | Serious |
| Mulvaney 2018[76] USA | Diabetes | 44 | Adolescent and young adult | RCT | App, Text messaging, Bluetooth-enabled glucometer | Patient | Blood glucose monitoring, diabetes self-management | No | Self-monitoring of behavior Feedback on behavior Prompts/cues Social reward | Mixed results | Some Concerns |
| Offringa 2018[77] USA | Diabetes | 1799 | Adult | Retrospective study | App | Patient | Taking medicine, Diet, and Exercise/physical therapy | No | Feedback on outcomes of behavior Self-monitoring of behavior Prompts/cues Self-monitoring of outcomes of behavior | Positive effect | Serious |
| Osborn 2020[78] USA | Diabetes | 95 | Adult | RCT | App, integrated glucometer, and activity tracker | Patient | Diabetes self-management | No | Self-monitoring of behavior Prompts/cues Feedback on behavior Goal setting (behavior) Feedback on outcomes of behavior Credible source Social support (unspecified) | Positive effect | Some Concerns |
| Osborn 2017[79] USA and non-USA (locations not further specified) | Diabetes | 1288 | Adult | Non-randomized experimental design | App, integrated glucometer | Patient | Taking medicine, Diet, Exercise/physical therapy, blood glucose monitoring | No | Self-monitoring of behavior Prompts/cues Feedback on behavior Goal setting (behavior) Feedback on outcomes of behavior Credible source Social support (unspecified) Self-monitoring of outcomes of behavior | Positive effect | Critical |
| Osborn 2017[80] USA and non-USA (locations not further specified) | Diabetes | 256 | Adult | Non-randomized experimental design | App, integrated glucometer | Patient | Taking medicine, Diet, Exercise/physical therapy, blood glucose monitoring | No | Self-monitoring of behavior Prompts/cues Feedback on behavior Goal setting (behavior) Feedback on outcomes of behavior Credible source Social support (unspecified) Self-monitoring of outcomes of behavior | Positive effect | Critical |
| Pramanik 2019[81] India | Diabetes | 34 | Adolescent and young adult | Non-randomized experimental design | App | Patient | Taking medicine, Diet, and Exercise/physical therapy | No | Prompts/cues Self-monitoring of outcomes of behavior | Positive effect | Serious |
| Raiff 2016[82] USA | Diabetes | 41 | Adolescent and young adult | RCT | Website, USB-connected glucometer | Patient | Blood glucose monitoring | No | Self-monitoring of behavior Instruction on how to perform the behavior Social support (emotional) Goal setting (behavior) Material reward (behavior) | Mixed results | High |
| Shamanna 2020[83] India | Diabetes | 64 | Adult | Non-randomized experimental design | App and self-monitoring devices integrated into app (activity sensor, glucometer, blood pressure meter, and scale) | Patient, Healthcare provider | Diet, Exercise/physical therapy, and blood glucose monitoring | No | Self-monitoring of behavior Feedback on behavior Action planning Social support (unspecified) Self-monitoring of outcomes of behavior Feedback on outcomes of behavior | Positive effect | Serious |
| Sittig 2020[84] USA | Diabetes | 20 | Adult | RCT | App and Text messaging | Patient | Diet, Exercise/physical therapy, diabetes self-management | Yes | Goal setting (behavior) Credible source Self-monitoring of behavior Prompts/cues Self-monitoring of outcomes of behavior | Positive effect | Some Concerns |
| Skrøvseth 2015[85] Norway | Diabetes | 30 | Adult | RCT | App, Bluetooth-enabled glucometer | Patient | Glycemic control | No | Self-monitoring of outcomes of behavior Feedback on outcomes of behavior | No effect | Some Concerns |
| Stanger 2018[86] USA | Diabetes | 61 | Adolescent and young adult | RCT | Website, Text messaging, Email, Phone | Patient, Caregiver, Healthcare provider | Blood glucose monitoring | Theory mentioned, unclear if it informed intervention | Social support (unspecified) Goal setting (behavior) Social support (practical) Material reward (behavior) Information about health consequences Salience of consequences Information about Antecedents Body changes Self-monitoring of behavior Feedback on behavior Pros and cons Reduce negative emotions Behavioral practice/rehearsal Behavioral contract Problem solving Instruction on how to perform the behavior | Positive effect | Some Concerns |
| Toschi 2018[87] USA | Diabetes | 30 | Adult | Non-randomized experimental design | App, Website, glucose sensor | Patient, Healthcare provider | Taking medicine, Diet, and blood glucose monitoring | No | Information about health consequences Feedback on outcomes of behavior Self-monitoring of behavior Self-monitoring of outcomes of behavior Prompts/cues Action planning Problem solving Feedback on behavior | Positive effect | Serious |
| Vinitha 2019[88] India | Diabetes | 248 | Adult | RCT | Text messaging | Patient | Diabetes self-management | No | Prompts/cues Instruction on how to perform the behavior | Positive effect | Some Concerns |
| Wang 2019[89] China | Diabetes | 120 | Adult | RCT | App | Patient, Healthcare provider | Diabetes self-management | No | Feedback on behavior Self-monitoring of behavior Prompts/cues Instruction on how to perform the behavior Credible source Social support (unspecified) Self-monitoring of outcomes of behavior Feedback on outcomes of behavior | Positive effect | Some Concerns |
| Yang 2020[90] South Korea | Diabetes | 247 | Adult | RCT | App | Patient, Healthcare provider | Blood glucose monitoring | No | Self-monitoring of outcomes of behavior Feedback on outcomes of behavior | Positive effect | High |
| Zhang 2018[91] USA | Diabetes | 48 | Adolescent and young adult | RCT | Text messaging | Patient, Caregiver | Glycemic control | Theory mentioned, unclear if it informed intervention | Social support (emotional) Prompts/cues | Mixed results | High |
| Lewinski 2019[92] USA | Diabetes/ Hypertension | 118 | Adult | Non-randomized experimental design | Text messaging, phone | Patient | Diabetes and hypertension self-management | Yes | Credible source Feedback on outcomes of behavior Conserving mental resources Prompts/cues | No effect | Serious |
| Guven 2020[93] Turkey | Epilepsy | 69 | Child, Adolescent and young adult | RCT | Text messaging and Website | Patient, Caregiver | Taking medicine | No | Credible source Prompts/cues | Positive effect | Some Concerns |
| Modi 2016[94] USA | Epilepsy | 25 | Adolescent and young adult | RCT | App and Text messaging | Patient, Caregiver | Taking medicine | No | Prompts/cues | Mixed results | High |
| Si 2020[95] China | Epilepsy | 380 | Adult | RCT | App | Patient, Healthcare provider | Epilepsy self-management | No | Self-monitoring of outcomes of behavior Credible source Feedback on outcomes of behavior Prompts/cues | Positive effect | Some Concerns |
| Christopolous 2018[96] USA | HIV | 230 | Adult | RCT | Text messaging | Patient | HIV self-management, viral suppression | Theory mentioned, unclear if it informed intervention | Prompts/cues | No effect | Low |
| Cook 2015[97] USA | HIV | 37 | Adult | RCT | Text messaging | Patient | Taking medicine | Theory mentioned, unclear if it informed intervention | Prompts/cues Self-monitoring of behavior Feedback on behavior | Mixed results | High |
| Cote 2015[98] USA | HIV | 88 | Adult | Non-randomized experimental design | Website | Patient | Taking medicine | Yes | Problem solving Self-monitoring of behavior Social support (emotional) Reduce negative emotions Instruction on how to perform the behavior | No effect | Low Concerns |
| Cote 2020[99] Canada | HIV | 179 | Adult | RCT | Website | Patient | Taking medicine | Theory mentioned, unclear if it informed intervention | Instruction on how to perform the behavior Reduce negative emotions Problem solving Social support (unspecified) Feedback on outcomes of behavior | No effect | High |
| Escobar-Viera 2020[100] USA | HIV | 132 | Adult | Non-randomized experimental design | App | Patient | Taking medicine | No | Prompts/cues Self-monitoring of behavior Feedback on behavior | Mixed results | Serious |
| Liran 2019[101] USA | HIV | 79 | Adult | Non-randomized experimental design | Interactive 3-dimensional educational VR experience | Patient | Taking medicine | No | Information about health consequences | Mixed results | Serious |
| Pagan-Ortiz 2019[102] USA | HIV | 21 | Adult | Mixed Methods | Text messaging | Patient | Taking medicine | Yes | Reduce prompts/cues Instruction on how to perform the behavior Information about health consequences Social support (unspecified) | Positive effect | Serious |
| Schnall 2018[103] USA | HIV | 80 | Adult | RCT | App | Patient | Taking medicine and HIV self-care | No | Instruction on how to perform the behavior Self-monitoring of outcomes of behavior Feedback on outcomes of behavior Prompts/cues | Positive effect | Some Concerns |
| Sherman 2020[104] USA | HIV | 94 | Adult | RCT | Text messaging | Patient | Taking medicine | No | Prompts/cues | No effect | Some Concerns |
| Whitely 2018[105] USA | HIV | 61 | Adolescent and young adult | RCT | App | Patient | Taking medicine and HIV self-management | Yes | Instruction on how to perform the behavior Social support (unspecified) Information about health consequences | Mixed results | Some Concerns |
| Zurlo 2020[106] USA | HIV | 92 | Adult | Non-randomized experimental design | App | Patient | Taking medicine, attending appointments and labs, viral suppression | No | Self-monitoring of outcomes of behavior Feedback on outcomes of behavior Goal setting (behavior) Prompts/cues Credible source | Positive effect | Serious |
| Carlsen 2017[107] Denmark | IBD/CD/UC | 50 | Child, Adolescent and young adult | Non-randomized experimental design | App | Patient, Caregiver, Healthcare provider | Symptom monitoring and stool sampling | No | Self-monitoring of outcomes of behavior Feedback on outcomes of behavior Prompts/cues Action planning | Positive effect | Serious |
| Carlsen 2017[108] Denmark | IBD/CD/UC | 53 | Child, Adolescent and young adult | RCT | Website | Patient, Caregiver, Healthcare provider | Symptom monitoring and stool sampling | No | Self-monitoring of outcomes of behavior Feedback on outcomes of behavior Prompts/cues Action planning | Mixed results | Some Concerns |
| Chudy-Onwugaje 2018[109] USA | IBD/CD/UC | 183 | Adult | RCT | Text messaging | Patient, Healthcare provider | IBD self-testing | No | Prompts/cues Self-monitoring of outcomes of behavior Feedback on outcomes of behavior | Mixed results | High |
| Cross 2019[110] USA | IBD/CD/UC | 348 | Adult | RCT | Text messaging | Patient, Healthcare provider | Disease activity | No | Prompts/cues Feedback on outcomes of behavior Action planning Self-monitoring of outcomes of behavior | Mixed results | Some Concerns |
| de Jong 2017[111] the Netherlands | IBD/CD/UC | 909 | Adult | RCT | Website | Patient, Healthcare provider | Taking medicine, disease activity, adherence to treatment | No | Self-monitoring of outcomes of behavior Self-monitoring of behavior Feedback on outcomes of behavior | Positive effect | Some Concerns |
| Del Hoyo 2018[112] Spain | IBD/CD/UC | 63 | Adult | RCT | App and Website | Patient, Healthcare provider | Taking medicine and disease activity | No | Self-monitoring of outcomes of behavior Feedback on outcomes of behavior Information about health consequences Prompts/cues Action planning | Mixed results | Some Concerns |
| McCombie 2020[113] New Zealand | IBD/CD/UC | 100 | Adult | RCT | App | Patient | Symptom and fecal calprotectin monitoring | No | Self-monitoring of outcomes of behavior Monitoring of outcomes of behavior without feedback | Mixed results | Some Concerns |
| Eaton 2020[114] USA | Kidney Disease | 34 | Adolescent and young adult | Study 1: RCT  Study 2: Non-randomized experimental design | Text messaging | Patient | Taking medicine | Yes | Prompts/cues Instruction on how to perform the behavior Information about health consequences | Mixed results | Study 1: Some Concerns  Study 2: Low |
| Min 2020[115] South Korea | Kidney Disease | 56 | Adult | Non-randomized experimental design | App | Patient | Taking medicine, Diet, and Exercise/physical therapy | No | Feedback on behavior Self-monitoring of outcomes of behavior Prompts/cues Instruction on how to perform the behavior Credible source | Mixed results | Serious |
| Anderson 2018[116] USA | Sickle cell disease | 32 | Child, Adolescent and young adult | Non-randomized experimental design | App | Patient | Taking medicine | No | Self-monitoring of behavior Feedback on behavior Information about health consequences | Positive effect | Serious |
| Leonard 2017[117] USA | Sickle cell disease | 11 | Adolescent and young adult | Non-randomized experimental design | App | Patient | Taking medicine | No | Self-monitoring of behavior Feedback on behavior Information about health consequences | Mixed results | Serious |
| DeVito Dabbs 2016[118] USA | Solid organ transplant | 201 | Adult | RCT | App | Patient | Tracking symptoms, vital signs, lab assay values | Yes | Self-monitoring of outcomes of behavior Feedback on outcomes of behavior Prompts/cues | Positive effect | Some Concerns |
| Geramita 2020[119] USA | Solid organ transplant | 105 | Adult | RCT | App | Patient | Taking medicine, Diet, Exercise/physical therapy, tracking health indicators, attending clinic appointments, performing home spirometry, monitoring blood pressure, abstaining from tobacco use | Yes | Self-monitoring of outcomes of behavior Feedback on outcomes of behavior Prompts/cues | Mixed results | High |
| Han 2019[120] South Korea | Solid organ transplant | 136 | Child, Adolescent and young adult, Adult | RCT | App | Patient | Taking medicine | No | Prompts/cues Self-monitoring of behavior Information about health consequences Self-monitoring of outcomes of behavior Feedback on behavior | No effect | Some Concerns |
| Levine 2019[121] USA | Solid organ transplant | 108 | Adult | RCT | App, Pebble Smart watch | Patient | Taking medicine | No | Prompts/cues Credible source | No effect | High |
| McGillicuddy 2020[122] USA | Solid organ transplant | 80 | Adult | RCT | App, Text messaging, Phone Calls, and electronic pill box | Patient, Healthcare provider | Taking medicine and blood pressure monitoring | Yes | Prompts/cues Self-monitoring of behavior Social support (unspecified) Monitoring of outcomes of behavior without feedback Self-monitoring of outcomes of behavior | Positive effect | Some Concerns |

**References for All Included Studies**

[[1]] Mary, Aurélien, Amélie Boursier, Isabelle Desailly Henry, Franck Grados, Alice Séjourné, Sarah Salomon, Patrice Fardellone, Michel Brazier, and Vincent Goëb. “Mobile Phone Text Messages and Effect on Treatment Adherence in Patients Taking Methotrexate for Rheumatoid Arthritis: A Randomized Pilot Study.” *Arthritis Care & Research* 71, no. 10 (2019): 1344–52. https://doi.org/10.1002/acr.23750.

[2] Ahmed, Sara, Pierre Ernst, Susan J Bartlett, Marie-France Valois, Tasneem Zaihra, Guy Paré, Roland Grad, Owis Eilayyan, Robert Perreault, and Robyn Tamblyn. “The Effectiveness of Web-Based Asthma Self-Management System, My Asthma Portal (MAP): A Pilot Randomized Controlled Trial.” *Journal of Medical Internet Research* 18, no. 12 (December 1, 2016): e313. https://doi.org/10.2196/jmir.5866.

[3] Barrett, Meredith A., Olivier Humblet, Justine E. Marcus, Kelly Henderson, Ted Smith, Nemr Eid, J. Wesley Sublett, et al. “Effect of a Mobile Health, Sensor-Driven Asthma Management Platform on Asthma Control.” *Annals of Allergy, Asthma & Immunology* 119, no. 5 (2017): 415-421.e1. https://doi.org/10.1016/j.anai.2017.08.002.

[4] Bender, Bruce G., Peter J. Cvietusa, Glenn K. Goodrich, Ryan Lowe, Heather A. Nuanes, Cynthia Rand, Susan Shetterly, et al. “Pragmatic Trial of Health Care Technologies to Improve Adherence to Pediatric Asthma Treatment: A Randomized Clinical Trial.” *JAMA Pediatrics* 169, no. 4 (April 1, 2015): 317. https://doi.org/10.1001/jamapediatrics.2014.3280.

[5] Cook, Kevin A., Brian D. Modena, and Ronald A. Simon. “Improvement in Asthma Control Using a Minimally Burdensome and Proactive Smartphone Application.” *The Journal of Allergy and Clinical Immunology: In Practice* 4, no. 4 (2016): 730-737.e1. https://doi.org/10.1016/j.jaip.2016.03.005.

[6 ]Davis, Sharon Ruth, Dorian Peters, Rafael Alejandro Calvo, Susan M Sawyer, Juliet M Foster, and Lorraine D Smith. “A Consumer Designed Smartphone App for Young People with Asthma: Pilot of Engagement and Acceptability.” *Journal of Asthma* 58, no. 2 (February 1, 2021): 253–61. https://doi.org/10.1080/02770903.2019.1680997.

[7] Khusial, Rishi J., Persijn J. Honkoop, Omar Usmani, Marcia Soares, Andrew Simpson, Martyn Biddiscombe, Sally Meah, et al. “Effectiveness of MyAirCoach: A MHealth Self-Management System in Asthma.” *The Journal of Allergy and Clinical Immunology: In Practice* 8, no. 6 (2020): 1972-1979.e8. https://doi.org/10.1016/j.jaip.2020.02.018.

[8] Kim, Mi-Yeong, Suh-Young Lee, Eun-Jung Jo, Seung-Eun Lee, Min-Gyu Kang, Woo-Jung Song, Sae-Hoon Kim, et al. “Feasibility of a Smartphone Application Based Action Plan and Monitoring in Asthma.” *Asia Pacific Allergy* 6, no. 3 (2016): 174. https://doi.org/10.5415/apallergy.2016.6.3.174.

[9] Kosse, Richelle C., Marcel L. Bouvy, Tjalling W. de Vries, and Ellen S. Koster. “Effect of a MHealth Intervention on Adherence in Adolescents with Asthma: A Randomized Controlled Trial.” *Respiratory Medicine* 149 (2019): 45–51. https://doi.org/10.1016/j.rmed.2019.02.009.

[10] Kosse, Richelle C, Marcel L Bouvy, Svetlana V Belitser, Tjalling W de Vries, Piet S van der Wal, and Ellen S Koster. “Effective Engagement of Adolescent Asthma Patients With Mobile Health–Supporting Medication Adherence.” *JMIR MHealth and UHealth* 7, no. 3 (March 27, 2019): e12411. https://doi.org/10.2196/12411

[11] Koufopoulos, Justin T, Mark T Conner, Peter H Gardner, and Ian Kellar. “A Web-Based and Mobile Health Social Support Intervention to Promote Adherence to Inhaled Asthma Medications: Randomized Controlled Trial.” *Journal of Medical Internet Research* 18, no. 6 (June 13, 2016): e122. https://doi.org/10.2196/jmir.4963.

[12] Lin, Nancy Y., Rachelle R. Ramsey, James L. Miller, Karen M. McDowell, Nanhua Zhang, Kevin Hommel, and Theresa W. Guilbert. “Telehealth Delivery of Adherence and Medication Management System Improves Outcomes in Inner‐city Children with Asthma.” *Pediatric Pulmonology* 55, no. 4 (2020): 858–65. https://doi.org/10.1002/ppul.24623.

[13] Ljungberg, Henrik, Anna Carleborg, Hilmar Gerber, Christina Öfverström, Jakob Wolodarski, Faiza Menshi, Mikaela Engdahl, Marianne Eduards, and Björn Nordlund. “Clinical Effect on Uncontrolled Asthma Using a Novel Digital Automated Self-Management Solution: A Physician-Blinded Randomised Controlled Crossover Trial.” *European Respiratory Journal* 54, no. 5 (November 14, 2019): 1900983. https://doi.org/10.1183/13993003.00983-2019.

[14] Kolmodin MacDonell, Karen, Sylvie Naar, Wanda Gibson-Scipio, Phebe Lam, and Elizabeth Secord. “The Detroit Young Adult Asthma Project: Pilot of a Technology-Based Medication Adherence Intervention for African-American Emerging Adults.” *Journal of Adolescent Health* 59, no. 4 (2016): 465–71. https://doi.org/10.1016/j.jadohealth.2016.05.016.

[15 ]Mammen, Jennifer R., Judith D. Schoonmaker, James Java, Jill Halterman, Marc N. Berliant, Amber Crowley, Marina Reznik, et al. “Going Mobile with Primary Care: Smartphone-Telemedicine for Asthma Management in Young Urban Adults (TEAMS).” *Journal of Asthma* 59, no. 1 (January 2, 2022): 132–44. https://doi.org/10.1080/02770903.2020.1830413.

[16] Merchant, Rajan K., Rubina Inamdar, and Robert C. Quade. “Effectiveness of Population Health Management Using the Propeller Health Asthma Platform: A Randomized Clinical Trial.” *The Journal of Allergy and Clinical Immunology: In Practice* 4, no. 3 (2016): 455–63. https://doi.org/10.1016/j.jaip.2015.11.022.

[17] Mosnaim, Giselle, Hong Li, Molly Martin, DeJuran Richardson, Paula Jo Belice, Elizabeth Avery, Agustina Silberstein, et al. “A Tailored Mobile Health Intervention to Improve Adherence and Asthma Control in Minority Adolescents.” *The Journal of Allergy and Clinical Immunology: In Practice* 3, no. 2 (2015): 288-290.e1. https://doi.org/10.1016/j.jaip.2014.10.011.

[18 ]Mosnaim, Giselle S., David A. Stempel, Candy Gonzalez, Brittany Adams, Naomi BenIsrael-Olive, Rahul Gondalia, Leanne Kaye, Madeleine Shalowitz, and Stanley Szefler. “The Impact of Patient Self-Monitoring Via Electronic Medication Monitor and Mobile App Plus Remote Clinician Feedback on Adherence to Inhaled Corticosteroids: A Randomized Controlled Trial.” *The Journal of Allergy and Clinical Immunology: In Practice* 9, no. 4 (2021): 1586–94. https://doi.org/10.1016/j.jaip.2020.10.064.

[19] Nemanic, Tiva, Irena Sarc, Sabina Skrgat, Matjaz Flezar, Iztok Cukjati, and Mateja Marc Malovrh. “Telemonitoring in Asthma Control: A Randomized Controlled Trial.” *Journal of Asthma* 56, no. 7 (July 3, 2019): 782–90. https://doi.org/10.1080/02770903.2018.1493599.

[20 ]Newhouse, Nikki, Angela Martin, Sena Jawad, Ly-Mee Yu, Mina Davoudianfar, Louise Locock, Sue Ziebland, and John Powell. “Randomised Feasibility Study of a Novel Experience-Based Internet Intervention to Support Self-Management in Chronic Asthma.” *BMJ Open* 6, no. 12 (2016): e013401. https://doi.org/10.1136/bmjopen-2016-013401.

[21] Perry, Tamara T., Alexandra Marshall, Ariel Berlinski, Mallikarjuna Rettiganti, Rita H. Brown, Shemeka M. Randle, Chunqiao Luo, and Jiang Bian. “Smartphone-Based vs Paper-Based Asthma Action Plans for Adolescents.” *Annals of Allergy, Asthma & Immunology* 118, no. 3 (2017): 298–303. https://doi.org/10.1016/j.anai.2016.11.028.

[22] Poureslami, Iraj, Jessica Shum, Richard T Lester, Hamid Tavakoli, Delbert R Dorscheid, and J. Mark FitzGerald. “A Pilot Randomized Controlled Trial on the Impact of Text Messaging Check-Ins and a Web-Based Asthma Action Plan versus a Written Action Plan on Asthma Exacerbations.” *Journal of Asthma* 56, no. 8 (October 16, 2018): 897–909. https://doi.org/10.1080/02770903.2018.1500583.

[23] Prabhakaran, Lathy, and Yap Chun Wei. “Effectiveness of the ECARE Programme: A Short Message Service for Asthma Monitoring.” *BMJ Health & Care Informatics* 26, no. 1 (2019): e100007. https://doi.org/10.1136/bmjhci-2019-100007.

[24] Real, Francis J., Andrew F. Beck, Dominick DeBlasio, Matthew Zackoff, Adrienne Henize, Yingying Xu, David Davis, Bradley Cruse, and Melissa D. Klein. “Dose Matters: A Smartphone Application to Improve Asthma Control Among Patients at an Urban Pediatric Primary Care Clinic.” *Games for Health Journal* 8, no. 5 (October 1, 2019): 357–65. https://doi.org/10.1089/g4h.2019.0011.

[25] Sleath, Betsy, Delesha Carpenter, Scott A. Davis, Charles Lee, Nacire Garcia, Daniel S. Reuland, Gail Tudor, and Ceila E. Loughlin. “The Impact of a Question Prompt List and Video Intervention on Teen Asthma Control and Quality-of-Life One Year Later: Results of a Randomized Trial.” *Journal of Asthma* 57, no. 9 (September 1, 2020): 1029–38. https://doi.org/10.1080/02770903.2019.1633542.

[26] Speck, Aimee L., Michael Hess, and Alan P. Baptist. “An Electronic Asthma Self-Management Intervention for Young African American Adults.” *The Journal of Allergy and Clinical Immunology: In Practice* 4, no. 1 (January 1, 2016): 89-95.e2. https://doi.org/10.1016/j.jaip.2015.08.007.

[27] Stukus, David R., Nabeel Farooqui, Kasey Strothman, Kelsey Ryan, Songzhu Zhao, Jack H. Stevens, and Daniel M. Cohen. “Real-World Evaluation of a Mobile Health Application in Children with Asthma.” *Annals of Allergy, Asthma & Immunology* 120, no. 4 (2018): 395-400.e1. https://doi.org/10.1016/j.anai.2018.02.006.

[28] Vasbinder, Erwin C., Lucas M.A. Goossens, Maureen P.M.H. Rutten-van Mölken, Brenda C.M. de Winter, Liset van Dijk, Arnold G. Vulto, Ellen I.M. Blankman, et al. “E-Monitoring of Asthma Therapy to Improve Compliance in Children (e-MATIC): A Randomised Controlled Trial.” *European Respiratory Journal* 48, no. 3 (2016): 758–67. https://doi.org/10.1183/13993003.01698-2015.

[29] Boon, M., J. Calvo-Lerma, I. Claes, T. Havermans, I. Asseiceira, A. Bulfamante, M. Garriga, et al. “Use of a Mobile Application for Self-Management of Pancreatic Enzyme Replacement Therapy Is Associated with Improved Gastro-Intestinal Related Quality of Life in Children with Cystic Fibrosis.” *Journal of Cystic Fibrosis* 19, no. 4 (2020): 562–68. https://doi.org/10.1016/j.jcf.2020.04.001.

[30] Cox, N. S., J. A. Alison, B. M. Button, J. W. Wilson, and A. E. Holland. “Feasibility and Acceptability of an Internet-Based Program to Promote Physical Activity in Adults With Cystic Fibrosis.” *Respiratory Care* 60, no. 3 (March 1, 2015): 422–29. https://doi.org/10.4187/respcare.03165.

[31] Drabble, Sarah J, Alicia O’Cathain, Alexander J Scott, Madelynne A Arden, Samuel Keating, Marlene Hutchings, Chin Maguire, and Martin Wildman. “Mechanisms of Action of a Web-Based Intervention With Health Professional Support to Increase Adherence to Nebulizer Treatments in Adults With Cystic Fibrosis: Qualitative Interview Study.” *Journal of Medical Internet Research* 22, no. 10 (October 6, 2020): e16782. https://doi.org/10.2196/16782.

[32] Floch, Jacqueline, Thomas Vilarinho, Annabel Zettl, Gema Ibanez-Sanchez, Joaquim Calvo-Lerma, Erlend Stav, Peter Halland Haro, Asbjørn Lein Aalberg, Alvaro Fides-Valero, and José Luis Bayo Montón. “Users’ Experiences of a Mobile Health Self-Management Approach for the Treatment of Cystic Fibrosis: Mixed Methods Study.” *JMIR MHealth and UHealth* 8, no. 7 (July 8, 2020): e15896. https://doi.org/10.2196/15896.

[33] Morton, Robert, Heather Elphick, Elaine Edwards, William J Daw, and Noreen S West. “Investigating the Feasibility of Text Message Reminders to Improve Adherence to Nebulized Medication in Children and Adolescents with Cystic Fibrosis.” *Patient Preference and Adherence* Volume 11 (2017): 861–69. https://doi.org/10.2147/PPA.S123723.

[34] Roehrer, Erin, Elizabeth Cummings, Leonie Ellis, and Paul Turner. “Value versus Use for Patients: Findings from an ICT Supported Cystic Fibrosis Self-Management Project,” n.d., 5.

[35] Adu, Mary D., Usman H. Malabu, Aduli E. O. Malau-Aduli, Aaron Drovandi, and Bunmi S. Malau-Aduli. “Efficacy and Acceptability of My Care Hub Mobile App to Support Self-Management in Australians with Type 1 or Type 2 Diabetes.” *International Journal of Environmental Research and Public Health* 17, no. 7 (April 9, 2020): 2573. https://doi.org/10.3390/ijerph17072573.

[36] Agarwal, Payal, Geetha Mukerji, Laura Desveaux, Noah M Ivers, Onil Bhattacharyya, Jennifer M Hensel, James Shaw, et al. “Mobile App for Improved Self-Management of Type 2 Diabetes: Multicenter Pragmatic Randomized Controlled Trial.” *JMIR MHealth and UHealth* 7, no. 1 (January 10, 2019): e10321. https://doi.org/10.2196/10321.

[37] Bajaj, Harpreet S., Karri Venn, Chenglin Ye, and Ronnie Aronson. “Randomized Trial of Long-Acting Insulin Glargine Titration Web Tool (LTHome) Versus Enhanced Usual Therapy of Glargine Titration (INNOVATE Trial).” *Diabetes Technology & Therapeutics* 18, no. 10 (2016): 610–15. https://doi.org/10.1089/dia.2016.0182.

[38] Boels, Anne Meike, Rimke C Vos, Lioe-Ting Dijkhorst-Oei, and Guy E H M Rutten. “Effectiveness of Diabetes Self-Management Education and Support via a Smartphone Application in Insulin-Treated Patients with Type 2 Diabetes: Results of a Randomized Controlled Trial (TRIGGER Study).” *BMJ Open Diabetes Research & Care* 7, no. 1 (2019): e000981. https://doi.org/10.1136/bmjdrc-2019-000981.

[39] Capozza, Korey, Sarah Woolsey, Mattias Georgsson, Jeff Black, Nelly Bello, Clare Lence, Steve Oostema, and Christie North. “Going Mobile With Diabetes Support: A Randomized Study of a Text Message–Based Personalized Behavioral Intervention for Type 2 Diabetes Self-Care.” *Diabetes Spectrum* 28, no. 2 (May 1, 2015): 83–91. https://doi.org/10.2337/diaspect.28.2.83.

[40] Castensøe-Seidenfaden, Pernille, Gitte Reventlov Husted, Andreas Kryger Jensen, Eva Hommel, Birthe Olsen, Ulrik Pedersen-Bjergaard, Finn Kensing, and Grete Teilmann. “Testing a Smartphone App (Young with Diabetes) to Improve Self-Management of Diabetes Over 12 Months: Randomized Controlled Trial.” *JMIR MHealth and UHealth* 6, no. 6 (June 26, 2018): e141. https://doi.org/10.2196/mhealth.9487.

[41] Chang, Hsiao-Yun, Huai-Lu Chang, An-Chi Chen, and Chiao-Hsuan Yen. “The Impact of M-Health on the Self- Management of Diabetes: A Preliminary Study,” n.d., 6.

[42] Chao, Dyna YP, Tom MY Lin, and Wen-Ya Ma. “Enhanced Self-Efficacy and Behavioral Changes Among Patients With Diabetes: Cloud-Based Mobile Health Platform and Mobile App Service.” *JMIR Diabetes* 4, no. 2 (May 10, 2019): e11017. https://doi.org/10.2196/11017.

[43] Chatzakis, Christos, Dimitrios Floros, Maria Papagianni, Kyriaki Tsiroukidou, Konstantina Kosta, Anastasios Vamvakis, Nikolaos Koletsos, Elpida Hatziagorou, Ioannis Tsanakas, and George Mastorakos. “The Beneficial Effect of the Mobile Application *Euglyca* in Children and Adolescents with Type 1 Diabetes Mellitus: A Randomized Controlled Trial.” *Diabetes Technology & Therapeutics* 21, no. 11 (November 1, 2019): 627–34. https://doi.org/10.1089/dia.2019.0170.

[44] Clements, Mark A, and Vincent S Staggs. “A Mobile App for Synchronizing Glucometer Data: Impact on Adherence and Glycemic Control Among Youths With Type 1 Diabetes in Routine Care.”*Journal of Diabetes Science and Technology* 11, no. 3 (2017): 461–67. https://doi.org/10.1177/1932296817691302.

[45 ]Davis, Scott A., Delesha Carpenter, Doyle M. Cummings, Charles Lee, Susan J. Blalock, Jennifer Elissa Scott, Lisa Rodebaugh, Stefanie P. Ferreri, and Betsy Sleath. “Patient Adoption of an Internet Based Diabetes Medication Tool to Improve Adherence: A Pilot Study.” *Patient Education and Counseling* 100, no. 1 (2017): 174–78. https://doi.org/10.1016/j.pec.2016.07.024.off

[46] Dobson, Rosie, Karen Carter, Richard Cutfield, Ashley Hulme, Richard Hulme, Catherine McNamara, Ralph Maddison, et al. “Diabetes Text-Message Self-Management Support Program (SMS4BG): A Pilot Study.” *JMIR MHealth and UHealth* 3, no. 1 (March 25, 2015): e32. https://doi.org/10.2196/mhealth.3988.

[47] Dobson, Rosie, Robyn Whittaker, Yannan Jiang, Ralph Maddison, Matthew Shepherd, Catherine McNamara, Richard Cutfield, Manish Khanolkar, and Rinki Murphy. “Effectiveness of Text Message Based, Diabetes Self Management Support Programme (SMS4BG): Two Arm, Parallel Randomised Controlled Trial.” *BMJ*, May 17, 2018, k1959. https://doi.org/10.1136/bmj.k1959.

[48] Garg, Satish K., Viral N. Shah, Halis K. Akturk, Christie Beatson, and Janet K. Snell-Bergeon. “Role of Mobile Technology to Improve Diabetes Care in Adults with Type 1 Diabetes: The Remote-T1D Study IBGStar® in Type 1 Diabetes Management.” *Diabetes Therapy* 8, no. 4 (2017): 811–19. https://doi.org/10.1007/s13300-017-0272-5.

[49] Gatwood, Justin, Rajesh Balkrishnan, Steven R. Erickson, Lawrence C. An, John D. Piette, and Karen B. Farris. “The Impact of Tailored Text Messages on Health Beliefs and Medication Adherence in Adults with Diabetes: A Randomized Pilot Study.” *Research in Social and Administrative Pharmacy* 12, no. 1 (2016): 130–40. https://doi.org/10.1016/j.sapharm.2015.04.007.

[50] Gimbel, Ronald W, Lior M Rennert, Paul Crawford, Jeanette R Little, Khoa Truong, Joel E Williams, Sarah F Griffin, et al. “Enhancing Patient Activation and Self-Management Activities in Patients With Type 2 Diabetes Using the US Department of Defense Mobile Health Care Environment: Feasibility Study.” *Journal of Medical Internet Research* 22, no. 5 (May 26, 2020): e17968. https://doi.org/10.2196/17968.

[51] Gong, Enying, Shaira Baptista, Anthony Russell, Paul Scuffham, Michaela Riddell, Jane Speight, Dominique Bird, Emily Williams, Mojtaba Lotfaliany, and Brian Oldenburg. “My Diabetes Coach, a Mobile App–Based Interactive Conversational Agent to Support Type 2 Diabetes Self-Management: Randomized Effectiveness-Implementation Trial.” *Journal of Medical Internet Research* 22, no. 11 (November 5, 2020): e20322. https://doi.org/10.2196/20322.

[52] Goyal, Shivani, Caitlin A Nunn, Michael Rotondi, Amy B Couperthwaite, Sally Reiser, Angelo Simone, Debra K Katzman, Joseph A Cafazzo, and Mark R Palmert. “A Mobile App for the Self-Management of Type 1 Diabetes Among Adolescents: A Randomized Controlled Trial.” *JMIR MHealth and UHealth* 5, no. 6 (June 19, 2017): e82. https://doi.org/10.2196/mhealth.7336.

[53] Gunawardena, Kasun C., Renee Jackson, Iva Robinett, Lahiru Dhaniska, Shaluka Jayamanne, Sumedha Kalpani, and Dimuthu Muthukuda. “The Influence of the Smart Glucose Manager Mobile Application on Diabetes Management.” *Journal of Diabetes Science and Technology* 13, no. 1 (2019): 75–81. https://doi.org/10.1177/1932296818804522.

[54] Guo, Sophie Huey-Ming, Her-Kun Chang, and Chun-Yi Lin. “Impact of Mobile Diabetes Self-Care System on Patients’ Knowledge, Behavior and Efficacy.” *Computers in Industry* 69 (2015): 22–29. https://doi.org/10.1016/j.compind.2014.11.001.

[55] Hannon, Tamara S, Lisa G Yazel-Smith, Amy S Hatton, Jennifer L Stanton, Elizabeth A S Moser, Xiaochun Li, and Aaron E Carroll. “Advancing Diabetes Management in Adolescents: Comparative Effectiveness of Mobile Self-Monitoring Blood Glucose Technology and Family-Centered Goal Setting.” *Pediatric Diabetes* 19, no. 4 (2018): 776–81. https://doi.org/10.1111/pedi.12648.

[56] Herbert, Linda Jones, Suzanne Collier, Alexa Stern, Maureen Monaghan, and Randi Streisand. “A Pilot Test of the Self-Management and Research Technology Project: A Text Message-Based Diabetes Self-Management Program for Adolescents.” *Journal of Child Health Care* 20, no. 4 (September 22, 2015): 456–63. https://doi.org/10.1177/1367493515603829.

[57] Hilliard, Marisa E., Viena T. Cao, Sahar S. Eshtehardi, Charles G. Minard, Rana Saber, Debbe Thompson, Lefkothea P. Karaviti, and Barbara J. Anderson. “Type 1 Doing Well: Pilot Feasibility and Acceptability Study of a Strengths-Based MHealth App for Parents of Adolescents with Type 1 Diabetes.” *Diabetes Technology & Therapeutics* 22, no. 11 (November 1, 2020): 835–45. https://doi.org/10.1089/dia.2020.0048.

[58] Hooshmandja, Manizhe, Aeen Mohammadi, Alireza Esteghamti, Khadije Aliabadi, and Mohammadreza Nili. “Effect of Mobile Learning (Application) on Self-Care Behaviors and Blood Glucose of Type 2 Diabetic Patients.” *Journal of Diabetes & Metabolic Disorders* 18, no. 2 (July 12, 2019): 307–13. https://doi.org/10.1007/s40200-019-00414-1.

[59] Jackson, Laura V., Diane M. Carpenter, Debbie A. Postlethwaite, Lorena C. Castro, Eileen Kim, and Ralph A. Herrera. “Evaluating the Impact of Mobile Phone Technology on Health Outcomes for Latinos with Type 2 Diabetes.” *Journal of Racial and Ethnic Health Disparities* 8, no. 2 (2021): 532–36. https://doi.org/10.1007/s40615-020-00810-x.

[60] Jeong, Ji Yun, Jae-Han Jeon, Kwi-Hyun Bae, Yeon-Kyung Choi, Keun-Gyu Park, Jung-Guk Kim, Kyu Chang Won, et al. “Smart Care Based on Telemonitoring and Telemedicine for Type 2 Diabetes Care: Multi-Center Randomized Controlled Trial.” *Telemedicine and E-Health* 24, no. 8 (2018): 604–13. https://doi.org/10.1089/tmj.2017.0203.

[61] Kaushal, Tara, Lorraine E Levitt Katz, Janet Joseph, Michelle Marowitz, Knashawn H Morales, Daniel Atkins, Dean Ritter, Reid Simon, Lori Laffel, and Terri H Lipman. “A Text Messaging Intervention With Financial Incentive for Adolescents With Type 1 Diabetes.” *Journal of Diabetes Science and Technology* 16, no. 1 (August 30, 2020): 120–27. https://doi.org/10.1177/1932296820952786.

[62] Kim, Eun Ky, Soo Heon Kwak, Seungsu Baek, Seung Lyeol Lee, Hak Chul Jang, Kyong Soo Park, and Young Min Cho. “Feasibility of a Patient-Centered, Smartphone-Based, Diabetes Care System: A Pilot Study.” *Diabetes & Metabolism Journal* 40, no. 3 (2016): 192. https://doi.org/10.4093/dmj.2016.40.3.192.

[63] Kim, Miyong T., Kim Byeng Kim, Tam H. Nguyen, Jisook Ko, Jim Zabora, Elizabeth Jacobs, and David Levine. “Motivating People to Sustain Healthy Lifestyles Using Persuasive Technology: A Pilot Study of Korean Americans with Prediabetes and Type 2 Diabetes.” *Patient Education and Counseling* 102, no. 4 (2019): 709–17. https://doi.org/10.1016/j.pec.2018.10.021.

[64] Kim, Eun Ky, Soo Heon Kwak, Hye Seung Jung, Bo Kyung Koo, Min Kyong Moon, Soo Lim, Hak Chul Jang, Kyong Soo Park, and Young Min Cho. “The Effect of a Smartphone-Based, Patient-Centered Diabetes Care System in Patients With Type 2 Diabetes: A Randomized, Controlled Trial for 24 Weeks.” *Diabetes Care* 42, no. 1 (2019): 3–9. https://doi.org/10.2337/dc17-2197.

[65] Kjos, Andrea L., Amy G. Vaughan, and Anuj Bhargava. “Impact of a Mobile App on Medication Adherence and Adherence-Related Beliefs in Patients with Type 2 Diabetes.” *Journal of the American Pharmacists Association* 59, no. 2 (2019): S44-S51.e3. https://doi.org/10.1016/j.japh.2018.12.012.

[66] Klee, Philippe, Catherine Bussien, Montserrat Castellsague, Christophe Combescure, Mirjam Dirlewanger, Celine Girardin, Jean-Luc Mando, et al. “An Intervention by a Patient-Designed Do-It-Yourself Mobile Device App Reduces HbA1c in Children and Adolescents with Type 1 Diabetes: A Randomized Double-Crossover Study.” *Diabetes Technology & Therapeutics* 20, no. 12 (December 4, 2018): 797–805. https://doi.org/10.1089/dia.2018.0255.

[67] Koot, David, Paul Soo Chye Goh, Robyn Su May Lim, Yubing Tian, Teng Yan Yau, Ngiap Chuan Tan, and Eric Andrew Finkelstein. “A Mobile Lifestyle Management Program (GlycoLeap) for People With Type 2 Diabetes: Single-Arm Feasibility Study.” *JMIR MHealth and UHealth* 7, no. 5 (May 24, 2019): e12965. https://doi.org/10.2196/12965.

[68] Ku, Eu Jeong, Ji‐In Park, Hyun Jeong Jeon, Taekeun Oh, and Hyung Jin Choi. “Clinical Efficacy and Plausibility of a Smartphone‐based Integrated Online Real‐time Diabetes Care System via Glucose and Diet Data Management: A Pilot Study.” *Internal Medicine Journal* 50, no. 12 (2020): 1524–32. https://doi.org/10.1111/imj.14738.

[69] Kumar, Shefali, Heidi Moseson, Jaspreet Uppal, and Jessie L Juusola. “A Diabetes Mobile App With In-App Coaching From a Certified Diabetes Educator Reduces A1C for Individuals With Type 2 Diabetes.” *The Diabetes Educator* 44, no. 3 (March 24, 2018): 225–36. https://doi.org/10.1177/0145721718765650.

[70] Lee, Da Young, Seung-Hyun Yoo, Kyong Pil Min, and Cheol-Young Park. “Effect of Voluntary Participation on Mobile Health Care in Diabetes Management: Randomized Controlled Open-Label Trial.” *JMIR MHealth and UHealth* 8, no. 9 (September 18, 2020): e19153. https://doi.org/10.2196/19153.

[71] Lim, Soo, Seon Mee Kang, Kyoung Min Kim, Jae Hoon Moon, Sung Hee Choi, Hee Hwang, Hye Seung Jung, Kyong Soo Park, Jun Oh Ryu, and Hak Chul Jang. “Multifactorial Intervention in Diabetes Care Using Real-Time Monitoring and Tailored Feedback in Type 2 Diabetes.” *Acta Diabetologica* 53, no. 2 (2016): 189–98. https://doi.org/10.1007/s00592-015-0754-8.

[72] Lin, Jian, Xia Li, Shan Jiang, Xiao Ma, Yuxin Yang, and Zhiguang Zhou. “Utilizing Technology-Enabled Intervention to Improve Blood Glucose Self-Management Outcome in Type 2 Diabetic Patients Initiated on Insulin Therapy: A Retrospective Real-World Study.” Edited by Peng Fei Shan. *International Journal of Endocrinology* 2020 (November 10, 2020): 1–8. https://doi.org/10.1155/2020/7249782.

[73] McGill, Dayna E., Lori M. Laffel, Lisa K. Volkening, Deborah A. Butler, Wendy L. Levy, Rachel M. Wasserman, and Barbara J. Anderson. “Text Message Intervention for Teens with Type 1 Diabetes Preserves HbA1c: Results of a Randomized Controlled Trial.” *Diabetes Technology & Therapeutics* 22, no. 5 (May 1, 2020): 374–82. https://doi.org/10.1089/dia.2019.0350.

[74] McLeod, Melissa, James Stanley, Virginia Signal, Jeannine Stairmand, Donna Thompson, Kelly Henderson, Cheryl Davies, et al. “Impact of a Comprehensive Digital Health Programme on HbA1c and Weight after 12 Months for People with Diabetes and Prediabetes: A Randomised Controlled Trial.” *Diabetologia* 63, no. 12 (2020): 2559–70. https://doi.org/10.1007/s00125-020-05261-x.

[75] Miller, Janice M., Ann G. Phalen, Albert Crawford, Anthony Frisby, and Barry S. Ziring. “Text Messaging in the Patient-Centered Medical Home to Improve Glucose Control and Retinopathy Screening.” *Health Equity* 1, no. 1 (2017): 2–6. https://doi.org/10.1089/heq.2016.0003.

[76] Mulvaney, Shelagh A., Sarah Vaala, Korey K. Hood, Cindy Lybarger, Rachel Carroll, Laura Williams, Douglas C. Schmidt, Kevin Johnson, Mary S. Dietrich, and Lori Laffel. “Mobile Momentary Assessment and Biobehavioral Feedback for Adolescents with Type 1 Diabetes: Feasibility and Engagement Patterns.” *Diabetes Technology & Therapeutics* 20, no. 7 (2018): 465–74. https://doi.org/10.1089/dia.2018.0064.

[77] Offringa, Reid, Tong Sheng, Linda Parks, Mark Clements, David Kerr, and Michael S. Greenfield. “Digital Diabetes Management Application Improves Glycemic Outcomes in People With Type 1 and Type 2 Diabetes.” *Journal of Diabetes Science and Technology* 12, no. 3 (2018): 701–8. https://doi.org/10.1177/1932296817747291.

[78] Osborn, Chandra Y, Ashley Hirsch, Lindsay E Sears, Mark Heyman, Jennifer Raymond, Brian Huddleston, and Jeff Dachis. “One Drop App With an Activity Tracker for Adults With Type 1 Diabetes: Randomized Controlled Trial.” *JMIR MHealth and UHealth* 8, no. 9 (September 17, 2020): e16745. https://doi.org/10.2196/16745.

[79] Osborn, Chandra Y, Joost R van Ginkel, David Rodbard, Mark Heyman, David G Marrero, Brian Huddleston, and Jeff Dachis. “One Drop | Mobile: An Evaluation of Hemoglobin A1c Improvement Linked to App Engagement.” *JMIR Diabetes* 2, no. 2 (August 24, 2017): e21. https://doi.org/10.2196/diabetes.8039.

[80] Osborn, Chandra Y, Joost R van Ginkel, David G Marrero, David Rodbard, Brian Huddleston, and Jeff Dachis. “One Drop | Mobile on IPhone and Apple Watch: An Evaluation of HbA1c Improvement Associated With Tracking Self-Care.” *JMIR MHealth and UHealth* 5, no. 11 (November 29, 2017): e179. https://doi.org/10.2196/mhealth.8781.

[81] Pramanik, Binay Kumar, J. Jebashini Angelin, Vineeth John Mathai, Sarah Mathai, Sophy Korula, and Anna Simon. “Smartphone App as Motivational Intervention to Improve Glycemic Control in Adolescents with Type 1 Diabetes.” *The Indian Journal of Pediatrics* 86, no. 12 (2019): 1118–23. https://doi.org/10.1007/s12098-019-03035-x.

[82] Raiff, Bethany R., Victoria B. Barrry, Ty A. Ridenour, and Natinee Jitnarin. “Internet-Based Incentives Increase Blood Glucose Testing with a Non-Adherent, Diverse Sample of Teens with Type 1 Diabetes Mellitus: A Randomized Controlled Trial.” *Translational Behavioral Medicine* 6, no. 2 (2016): 179–88. https://doi.org/10.1007/s13142-016-0397-5.

[83] Shamanna, Paramesh, Banshi Saboo, Suresh Damodharan, Jahangir Mohammed, Maluk Mohamed, Terrence Poon, Nathan Kleinman, and Mohamed Thajudeen. “Reducing HbA1c in Type 2 Diabetes Using Digital Twin Technology-Enabled Precision Nutrition: A Retrospective Analysis.” *Diabetes Therapy* 11, no. 11 (2020): 2703–14. https://doi.org/10.1007/s13300-020-00931-w.

[84] Sittig, Scott, Jing Wang, Sriram Iyengar, Sahiti Myneni, and Amy Franklin. “Incorporating Behavioral Trigger Messages Into a Mobile Health App for Chronic Disease Management: Randomized Clinical Feasibility Trial in Diabetes.” *JMIR MHealth and UHealth* 8, no. 3 (March 16, 2020): e15927. https://doi.org/10.2196/15927.

[85] Skrøvseth, Stein Olav, Eirik Årsand, Fred Godtliebsen, and Ragnar M. Joakimsen. “Data-Driven Personalized Feedback to Patients with Type 1 Diabetes: A Randomized Trial.” *Diabetes Technology & Therapeutics* 17, no. 7 (2015): 482–89. https://doi.org/10.1089/dia.2014.0276.

[86] Stanger, Catherine, Amy Hughes Lansing, Emily Scherer, Alan Budney, Ann S. Christiano, and Samuel J Casella. “A Web-Delivered Multicomponent Intervention for Adolescents with Poorly Controlled Type 1 Diabetes: A Pilot Randomized Controlled Trial.” *Annals of Behavioral Medicine* 52, no. 12 (November 12, 2018): 1010–22. https://doi.org/10.1093/abm/kay005.

[87] Toschi, Elena, Lawrence Fisher, Howard Wolpert, Michael Love, Timothy Dunn, and Gary Hayter. “Evaluating a Glucose-Sensor-Based Tool to Help Clinicians and Adults With Type 1 Diabetes Improve Self-Management Skills.” *Journal of Diabetes Science and Technology* 12, no. 6 (2018): 1143–51. https://doi.org/10.1177/1932296818791534.

[88] Vinitha, Ramachandran, Arun Nanditha, Chamukuttan Snehalatha, Krishnamoorthy Satheesh, Priscilla Susairaj, Arun Raghavan, and Ambady Ramachandran. “Effectiveness of Mobile Phone Text Messaging in Improving Glycaemic Control among Persons with Newly Detected Type 2 Diabetes.” *Diabetes Research and Clinical Practice* 158 (2019): 107919. https://doi.org/10.1016/j.diabres.2019.107919.

[89] Wang, Yanmei, Ming Li, Xinxiang Zhao, Xinxin Pan, Min Lu, Jing Lu, and Yan Hu. “Effects of Continuous Care for Patients with Type 2 Diabetes Using Mobile Health Application: A Randomised Controlled Trial.” *The International Journal of Health Planning and Management* 34, no. 3 (2019): 1025–35. https://doi.org/10.1002/hpm.2872.

[90] Yang, Yeoree, Eun Young Lee, Hun-Sung Kim, Seung-Hwan Lee, Kun-Ho Yoon, and Jae-Hyoung Cho. “Effect of a Mobile Phone–Based Glucose-Monitoring and Feedback System for Type 2 Diabetes Management in Multiple Primary Care Clinic Settings: Cluster Randomized Controlled Trial.” *JMIR MHealth and UHealth* 8, no. 2 (February 26, 2020): e16266. https://doi.org/10.2196/16266.

[91] Zhang, Shuodan, Emily Hamburger, Sachini Kahanda, Morgan Lyttle, Rodayne Williams, and Sarah S. Jaser. “Engagement with a Text-Messaging Intervention Improves Adherence in Adolescents with Type 1 Diabetes: Brief Report.” *Diabetes Technology & Therapeutics* 20, no. 5 (2018): 386–89. https://doi.org/10.1089/dia.2018.0015.

[92] Lewinski, Allison A., Uptal D Patel, Clarissa J Diamantidis, Megan Oakes, Khaula Baloch, Matthew J Crowley, Jonathan Wilson, et al. “Addressing Diabetes and Poorly Controlled Hypertension: Pragmatic MHealth Self-Management Intervention.” *Journal of Medical Internet Research* 21, no. 4 (April 9, 2019): e12541. https://doi.org/10.2196/12541.

[93] Tutar Güven, Şerife, Ayşegül İşler Dalgiç, and Özgür Duman. “Evaluation of the Efficiency of the Web-Based Epilepsy Education Program (WEEP) for Youth with Epilepsy and Parents: A Randomized Controlled Trial.” *Epilepsy & Behavior* 111 (2020): 107142. https://doi.org/10.1016/j.yebeh.2020.107142.

[94] Modi, Avani C., Krista A. Mann, Lauryn Urso, and James Peugh. “Preliminary Feasibility and Efficacy of Text Messaging and Application-Based Adherence Interventions in Adolescents with Epilepsy.” *Epilepsy & Behavior* 63 (2016): 46–49. https://doi.org/10.1016/j.yebeh.2016.07.036.

[95] Si, Yang, Xiaoqiang Xiao, Cai Xia, Jiang Guo, Qiukui Hao, Qianning Mo, Yulong Niu, and Hongbin Sun. “Optimising Epilepsy Management with a Smartphone Application: A Randomised Controlled Trial.” *Medical Journal of Australia* 212, no. 6 (2020): 258–62. https://doi.org/10.5694/mja2.50520.

[96] Christopoulos, Katerina A, Elise D Riley, Adam W Carrico, Jacqueline Tulsky, Judith T Moskowitz, Samantha Dilworth, Lara S Coffin, Leslie Wilson, Jason Johnson Peretz, and Joan F Hilton. “A Randomized Controlled Trial of a Text Messaging Intervention to Promote Virologic Suppression and Retention in Care in an Urban Safety-Net Human Immunodeficiency Virus Clinic: The Connect4Care Trial.” *Clinical Infectious Diseases* 67, no. 5 (August 16, 2018): 751–59. https://doi.org/10.1093/cid/ciy156.

[97] Cook, Paul F, Jane M Carrington, Sarah J Schmiege, Whitney Starr, and Blaine Reeder. “A Counselor in Your Pocket: Feasibility of Mobile Health Tailored Messages to Support HIV Medication Adherence.” *Patient Preference and Adherence*, September 23, 2015, 1353–66. https://doi.org/10.2147/PPA.S88222.

[98] Côté, José, Gaston Godin, Pilar Ramirez-Garcia, Geneviève Rouleau, Anne Bourbonnais, Yann-Gaël Guéhéneuc, Cécile Tremblay, and Joanne Otis. “Virtual Intervention to Support Self-Management of Antiretroviral Therapy Among People Living With HIV.” *Journal of Medical Internet Research* 17, no. 1 (January 6, 2015): e6. https://doi.org/10.2196/jmir.3264.

[99] Côté, José, Geneviève Rouleau, Maria Pilar Ramirez-Garcia, Patricia Auger, Réjean Thomas, and Judith Leblanc. “Effectiveness of a Web-Based Intervention to Support Medication Adherence Among People Living With HIV: Web-Based Randomized Controlled Trial.” *JMIR Public Health and Surveillance* 6, no. 2 (April 20, 2020): e17733. https://doi.org/10.2196/17733.

[100] Escobar-Viera, César, Zhi Zhou, Jamie P Morano, Robert Lucero, Spencer Lieb, Sean McIntosh, Kevin A Clauson, and Robert L Cook. “The Florida Mobile Health Adherence Project for People Living With HIV (FL-MAPP): Longitudinal Assessment of Feasibility, Acceptability, and Clinical Outcomes.” *JMIR MHealth and UHealth* 8, no. 1 (January 8, 2020): e14557. https://doi.org/10.2196/14557.

[101] Liran, Omer, Robert Dasher, and Kevin Kaeochinda. “Using Virtual Reality to Improve Antiretroviral Therapy Adherence in the Treatment of HIV: Open-Label Repeated Measure Study.” *Interactive Journal of Medical Research* 8, no. 2 (June 20, 2019): e13698. https://doi.org/10.2196/13698.

[102] Pagan-Ortiz, Marta E., Paul Goulet, Laura Kogelman, Sue E. Levkoff, and Patricia Flynn Weitzman. “Feasibility of a Texting Intervention to Improve Medication Adherence Among Older HIV+ African Americans: A Mixed-Method Pilot Study.” *Gerontology and Geriatric Medicine* 5 (2019): 233372141985566. https://doi.org/10.1177/2333721419855662.

[103] Schnall, Rebecca, Hwayoung Cho, Alexander Mangone, Adrienne Pichon, and Haomiao Jia. “Mobile Health Technology for Improving Symptom Management in Low Income Persons Living with HIV.” *AIDS and Behavior* 22, no. 10 (2018): 3373–83. https://doi.org/10.1007/s10461-017-2014-0.

[104] Sherman, Elizabeth M., Jianli Niu, Shara Elrod, Kevin A. Clauson, Fadi Alkhateeb, and Paula Eckardt. “Effect of Mobile Text Messages on Antiretroviral Medication Adherence and Patient Retention in Early HIV Care: An Open-Label, Randomized, Single Center Study in South Florida.” *AIDS Research and Therapy* 17, no. 1 (May 13, 2020): 16. https://doi.org/10.1186/s12981-020-00275-2.

[105] Whiteley, Laura, Larry K. Brown, Leandro Mena, Lacey Craker, and Trisha Arnold. “Enhancing Health among Youth Living with HIV Using an IPhone Game.” *AIDS Care* 30, no. sup4 (July 25, 2018): 21–33. https://doi.org/10.1080/09540121.2018.1503224.

[106] Zurlo, John, Ping Du, Alexander Haynos, Verbenia Collins, Tarek Eshak, and Cynthia Whitener. “OPT-In For Life: A Mobile Technology–Based Intervention to Improve HIV Care Continuum for Young Adults Living With HIV.” *Health Promotion Practice* 21, no. 5 (2020): 727–37. https://doi.org/10.1177/1524839920936247.

[107] Carlsen, Katrine, Gunnar Houen, Christian Jakobsen, Thomas Kallemose, Anders Paerregaard, Lene B. Riis, Pia Munkholm, and Vibeke Wewer. “Individualized Infliximab Treatment Guided by Patient-Managed EHealth in Children and Adolescents with Inflammatory Bowel Disease:” *Inflammatory Bowel Diseases* 23, no. 9 (2017): 1473–82. https://doi.org/10.1097/MIB.0000000000001170.

[108] Carlsen, Katrine, Christian Jakobsen, Gunnar Houen, Thomas Kallemose, Anders Paerregaard, Lene B. Riis, Pia Munkholm, and Vibeke Wewer. “Self-Managed EHealth Disease Monitoring in Children and Adolescents with Inflammatory Bowel Disease: A Randomized Controlled Trial.” *Inflammatory Bowel Diseases*, 2017, 1. https://doi.org/10.1097/MIB.0000000000001026.

[109] Chudy-Onwugaje, Kenechukwu, Ameer Abutaleb, Andrea Buchwald, Patricia Langenberg, Miguel Regueiro, David A Schwartz, J Kathleen Tracy, et al. “Age Modifies the Association Between Depressive Symptoms and Adherence to Self-Testing With Telemedicine in Patients With Inflammatory Bowel Disease.” *Inflammatory Bowel Diseases* 24, no. 12 (November 29, 2018): 2648–54. https://doi.org/10.1093/ibd/izy194.

[110] Cross, Raymond K, Patricia Langenberg, Miguel Regueiro, David A. Schwartz, J. Kathleen Tracy, Joseph F Collins, Jonathan Katz, et al. “A Randomized Controlled Trial of TELEmedicine for Patients with Inflammatory Bowel Disease (TELE-IBD).” *American Journal of Gastroenterology* 114, no. 3 (2019): 472–82. https://doi.org/10.1038/s41395-018-0272-8.

[111] Jong, Marin J de, Andrea E van der Meulen-de Jong, Mariëlle J Romberg-Camps, Marco C Becx, Jeroen P Maljaars, Mia Cilissen, Ad A van Bodegraven, et al. “Telemedicine for Management of Inflammatory Bowel Disease (MyIBDcoach): A Pragmatic, Multicentre, Randomised Controlled Trial.” *The Lancet* 390, no. 10098 (2017): 959–68. https://doi.org/10.1016/S0140-6736(17)31327-2.

[112] Del Hoyo, Javier, Pilar Nos, Raquel Faubel, Diana Muñoz, David Domínguez, Guillermo Bastida, Bernardo Valdivieso, Marisa Correcher, and Mariam Aguas. “A Web-Based Telemanagement System for Improving Disease Activity and Quality of Life in Patients With Complex Inflammatory Bowel Disease: Pilot Randomized Controlled Trial.” *Journal of Medical Internet Research* 20, no. 11 (November 27, 2018): e11602. https://doi.org/10.2196/11602.

[113] McCombie, Andrew, Russell Walmsley, Murray Barclay, Christine Ho, Tobias Langlotz, Holger Regenbrecht, Andrew Gray, Nideen Visesio, Stephen Inns, and Michael Schultz. “A Noninferiority Randomized Clinical Trial of the Use of the Smartphone-Based Health Applications IBDsmart and IBDoc in the Care of Inflammatory Bowel Disease Patients.” *Inflammatory Bowel Diseases* 26, no. 7 (2020): 1098–1109. https://doi.org/10.1093/ibd/izz252.

[114] Eaton, Cyd, Margaret Comer, Cozumel Pruette, Kevin Psoter, and Kristin Riekert. “Text Messaging Adherence Intervention for Adolescents and Young Adults with Chronic Kidney Disease: Pilot Randomized Controlled Trial and Stakeholder Interviews.” *Journal of Medical Internet Research* 22, no. 8 (August 14, 2020): e19861. https://doi.org/10.2196/19861.

[115] Min, Youngsoon, and Myonghwa Park. “Effects of a Mobile-App-Based Self-Management Support Program For Elderly Hemodialysis Patients.” *Healthcare Informatics Research* 26, no. 2 (April 30, 2020): 93–103. https://doi.org/10.4258/hir.2020.26.2.93.

[116] Anderson, Lindsay M., Sarah Leonard, Jude Jonassaint, Joseph Lunyera, Melanie Bonner, and Nirmish Shah. “Mobile Health Intervention for Youth with Sickle Cell Disease: Impact on Adherence, Disease Knowledge, and Quality of Life.” *Pediatric Blood & Cancer* 65, no. 8 (2018): e27081. https://doi.org/10.1002/pbc.27081.

[117] Leonard, Sarah, Lindsay M. Anderson, Jude Jonassaint, Charles Jonassaint, and Nirmish Shah. “Utilizing a Novel Mobile Health ‘Selfie’ Application to Improve Compliance to Iron Chelation in Pediatric Patients Receiving Chronic Transfusions.” *Journal of Pediatric Hematology/Oncology* 39, no. 3 (2017): 223–29. https://doi.org/10.1097/MPH.0000000000000743.

[118] DeVito Dabbs, A, M K Song, B A Myers, R Li, R P Hawkins, J M Pilewski, C A Bermudez, et al. “A Randomized Controlled Trial of a Mobile Health Intervention to Promote Self-Management After Lung Transplantation.” *American Journal of Transplantation* 16, no. 7 (2016): 2172–80. https://doi.org/10.1111/ajt.13701.

[119] Geramita, Emily M., Annette J. DeVito Dabbs, Andrea F. DiMartini, Joseph M. Pilewski, Galen E. Switzer, Donna M. Posluszny, Larissa Myaskovsky, and Mary Amanda Dew. “Impact of a Mobile Health Intervention on Long-Term Nonadherence After Lung Transplantation: Follow-up After a Randomized Controlled Trial.” *Transplantation* 104, no. 3 (2020): 640–51. https://doi.org/10.1097/TP.0000000000002872.

[120] Han, Ahram, Sang-il Min, Sanghyun Ahn, Seung-Kee Min, Hye-jin Hong, Nayoung Han, Yon Su Kim, Curie Ahn, and Jongwon Ha. “Mobile Medication Manager Application to Improve Adherence with Immunosuppressive Therapy in Renal Transplant Recipients: A Randomized Controlled Trial.” Edited by Shahrad Taheri. *PLOS ONE* 14, no. 11 (November 5, 2019): e0224595. https://doi.org/10.1371/journal.pone.0224595.

[121] Levine, Dov, Julia Torabi, Krystina Choinski, Juan P. Rocca, and Jay A. Graham. “Transplant Surgery Enters a New Era: Increasing Immunosuppressive Medication Adherence through Mobile Apps and Smart Watches.” *The American Journal of Surgery* 218, no. 1 (2019): 18–20. https://doi.org/10.1016/j.amjsurg.2019.02.018.

[122] McGillicuddy, John W., Jessica L. Chandler, Luke R. Sox, and David J. Taber. “Exploratory Analysis of the Impact of an MHealth Medication Adherence Intervention on Tacrolimus Trough Concentration Variability: Post Hoc Results of a Randomized Controlled Trial.” *Annals of Pharmacotherapy* 54, no. 12 (2020): 1185–93. https://doi.org/10.1177/1060028020931806.

**TABLE S2. ADHERENCE MEASURE TYPES TABLE**

Type of Adherence or Self-Management Outcome Measurement in All Included Studies (Overall), Adult Only Studies, and Adolescent/Young Adult Only Studies and by Study Results Direction

|  | **Overall** | | | | | | | | | **Adult Only** | | | | | | | | **Adolescent/Young Adult Only** | | | | | | | |
| --- | --- | --- | --- | --- | --- | --- | --- | --- | --- | --- | --- | --- | --- | --- | --- | --- | --- | --- | --- | --- | --- | --- | --- | --- | --- |
|  | Overall Total | | Positive | | No Impact | | Mixed | | Adult Total | | | Positive | | No Impact | | Mixed | | Adolescent/ Young Adult Total | | Positive | | No Impact | | Mixed | |
|  | n=122 | | n=59 | | n=23 | | n=40 | | n=81 | | | n=45 | | n=18 | | n=18 | | n=22 | | n=4 | | n=2 | | n=16 | |
|  | n | % | n | % | n | % | n | % | n | | % | n | % | n | % | n | % | n | % | n | % | n | % | n | % |
| Objective Behavior | 35 | 29% | 20 | 34% | 3 | 13% | 12 | 30% | 18 | | 22% | 13 | 29% | 2 | 11% | 3 | 17% | 9 | 41% | 2 | 50% | 0 | 0% | 7 | 44% |
| Subjective Behavior | 61 | 50% | 28 | 47% | 10 | 43% | 23 | 58% | 40 | | 49% | 22 | 49% | 7 | 39% | 11 | 61% | 10 | 45% | 1 | 25% | 0 | 0% | 9 | 56% |
| Psychosocial Factors | 2 | 2% | 0 | 0% | 0 | 0% | 2 | 5% | 0 | | 0% | 0 | 0% | 0 | 0% | 0 | 0% | 2 | 9% | 0 | 0% | 0 | 0% | 2 | 13% |
| Objective Health Outcomes | 61 | 50% | 32 | 54% | 13 | 57% | 16 | 40% | 44 | | 54% | 27 | 60% | 10 | 56% | 7 | 39% | 12 | 55% | 2 | 50% | 2 | 100% | 8 | 50% |
| Subjective Health Outcomes | 29 | 24% | 12 | 20% | 6 | 26% | 11 | 28% | 17 | | 21% | 8 | 18% | 4 | 22% | 5 | 28% | 4 | 18% | 0 | 0% | 0 | 0% | 4 | 25% |

*Note.* Percentages do not add up to 100% because studies could use more than one type of adherence/self-management measurement.

**TABLE S3. BEHAVIOR CHANGE TECHNIQUES DEFINITIONS TABLE**

Behavior Change Techniques in All Included Studies (Overall), Adult Only Studies, and Adolescent/Young Adult Only Studies and by Study Results Direction

|  | **Overall** | | | | | | | | **Adult Only** | | | | | | | | **Adolescent/Young Adult Only** | | | | | | | |
| --- | --- | --- | --- | --- | --- | --- | --- | --- | --- | --- | --- | --- | --- | --- | --- | --- | --- | --- | --- | --- | --- | --- | --- | --- |
|  | Overall Total  n=122 | | Positive  n=59/122, 48% | | No Impact  n=23/122, 19% | | Mixed  n=40/122, 33% | | Adult Total  n=81/122, 66% | | Positive  n=45/81, 56% | | No Impact  n=18/81, 22% | | Mixed  n=18/81, 22% | | Adolescent/Young Adult Total  n=22/122, 34% | | Positive  n=4/22, 18% | | No Impact  n=2/22, 9% | | Mixed  n=16/22, 73% | |
| **Average number of BCTs (M (SD))** | 4.30 (2.32) | | 4.95 (2.56) | | 3.57 (1.95) | | 3.9 (1.93) | | 4.47 (2.21) | | 5.02 (2.13) | | 3.28 (1.96) | | 4.28 (2.27) | | 4.41 (3.14) | | 7.75 (5.91) | | 5.00 (1.41) | | 3.50 (1.67) | |
| **Behavior change technique with examples** | n | % | n | % | n | % | n | % | n | % | n | % | n | % | n | % | n | % | n | % | n | % | n | % |
| **Prompts/cues***. Reminder to take medicine, test blood glucose· Push notification or text message sent to user* | 85 | 70 | 44 | 75 | 15 | 65 | 26 | 65 | 58 | 72 | 34 | 76 | 11 | 61 | 13 | 72 | 15 | 68 | 3 | 75 | 2 | 100 | 10 | 63 |
| **Self-monitoring of outcomes of behavior***. User tracks information about outcomes of behavior (e.g., HbA1c level, weight)*. *Manually entered in app or automatically uploaded via a device (e.g., Bluetooth-enabled glucometer or scale)* | 73 | 60 | 39 | 66 | 11 | 48 | 23 | 58 | 54 | 67 | 33 | 73 | 7 | 39 | 14 | 78 | 9 | 41 | 2 | 50 | 1 | 50 | 6 | 38 |
| **Feedback on outcomes of behavior***. Graphs of self-monitored outcomes of behavior displayed in app· Communication with care team (user can ask questions, receive feedback on self-monitored data)* | 60 | 49 | 34 | 58 | 9 | 39 | 17 | 43 | 48 | 59 | 30 | 67 | 7 | 39 | 11 | 61 | 5 | 23 | 1 | 25 | 1 | 50 | 3 | 19 |
| **Self-monitoring of behavior.** *User tracks information about behavior (e.g., taking medicine, exercising, self-testing blood glucose). Manually entered in app or automatically uploaded via a device (e.g., Bluetooth-enabled inhaler, pedometer)* | 56 | 46 | 34 | 58 | 8 | 35 | 14 | 35 | 39 | 48 | 26 | 58 | 6 | 33 | 7 | 39 | 8 | 36 | 3 | 75 | 0 | 0 | 5 | 31 |
| **Feedback on behavior.** *Graphs of self-monitored outcomes of behavior displayed in app*  *Communication with care team (user can ask questions, receive feedback on self-monitored data)* | 46 | 38 | 29 | 49 | 4 | 17 | 13 | 33 | 29 | 36 | 20 | 44 | 2 | 11 | 7 | 39 | 7 | 32 | 3 | 75 | 1 | 50 | 3 | 19 |
| **Credible source.**  *Informational content about chronic condition and self-management. Developed and delivered by experts* | 41 | 34 | 24 | 41 | 7 | 30 | 10 | 25 | 32 | 40 | 21 | 47 | 6 | 33 | 5 | 28 | 6 | 27 | 1 | 25 | 1 | 50 | 4 | 25 |
| **Social support (unspecified)*.*** *Social networking components; elements of cognitive behavioral therapy and motivational interviewing* | 27 | 22 | 16 | 27 | 6 | 26 | 5 | 13 | 20 | 25 | 14 | 31 | 4 | 22 | 2 | 11 | 6 | 27 | 2 | 50 | 1 | 50 | 3 | 19 |
| **Instruction on how to perform the behavior.** *Skills training (e.g., how to use an inhaler)* | 22 | 18 | 12 | 20 | 5 | 22 | 5 | 13 | 15 | 19 | 9 | 20 | 4 | 22 | 2 | 11 | 6 | 27 | 2 | 50 | 1 | 50 | 3 | 19 |
| **Goal setting (behavior).** *Set goal for number of times to exercise, log meals, take medicine, etc. in a specified period of time* | 19 | 16 | 14 | 24 | 1 | 4 | 4 | 10 | 16 | 20 | 13 | 29 | 1 | 6 | 2 | 11 | 3 | 14 | 1 | 25 | 0 | 0 | 2 | 13 |
| **Action planning***. Provide detailed plan for managing medical condition based on data logged in app* | 18 | 15 | 7 | 12 | 3 | 13 | 8 | 20 | 11 | 14 | 6 | 13 | 1 | 6 | 4 | 22 | 2 | 9 | 0 | 0 | 1 | 50 | 1 | 6 |
| **Information about health consequences.** *Provide information about positive health effects of taking a prescribed daily medication and potential negative consequences of not taking this medicine* | 16 | 13 | 6 | 10 | 2 | 9 | 8 | 20 | 7 | 9 | 4 | 9 | 1 | 6 | 2 | 11 | 5 | 23 | 1 | 25 | 0 | 0 | 4 | 25 |
| **Conserving mental resources.**  *Provides strategies to reduce the cognitive demands of behavior change (e.g., app contains a user-friendly calculator to recommend bolus insulin dose based on carbohydrate intake)* | 13 | 11 | 6 | 10 | 2 | 9 | 5 | 13 | 9 | 11 | 4 | 9 | 2 | 11 | 3 | 17 | 0 | 0 | 0 | 0 | 0 | 0 | 0 | 0 |
| **Problem solving.** *Select strategies to address barriers or to facilitate behavior changes (e.g., user reports on barriers to adherence receives text messages with tailored recommendations for overcoming these barriers)* | 9 | 7 | 6 | 10 | 2 | 9 | 1 | 3 | 6 | 7 | 4 | 9 | 2 | 11 | 0 | 0 | 2 | 9 | 1 | 25 | 0 | 0 | 1 | 6 |
| **Social support (emotional).** *Deliver emotion social support to encourage performance of a health behavior (e.g., user receives text messages with content related to managing emotional distress related to treatment recommendation)* | 6 | 5 | 2 | 3 | 1 | 4 | 3 | 8 | 3 | 4 | 2 | 4 | 1 | 6 | 0 | 0 | 3 | 14 | 0 | 0 | 0 | 0 | 3 | 19 |
| **Material reward (behavior).**  *Deliver a tangible reward (e.g., money) for completing a specified health behavior (e.g., user earns $1 each day that an electronic pill bottle records medication as "taken")* | 6 | 5 | 3 | 5 | 0 | 0 | 3 | 8 | 1 | 1 | 1 | 2 | 0 | 0 | 0 | 0 | 5 | 23 | 2 | 50 | 0 | 0 | 3 | 19 |

*Note.* Percentages do not add up to 100% because studies could use more than one type of behavior change technique.

**TABLE S4. BEHAVIOR CHANGE TECHNIQUES BY INTERVENTION TARGET**

|  | **Intervention Explicitly Targets Taking Medicine** | | | | | | | | **Intervention Targets Self-Management and Other Adherence Targets** | | | | | | | |
| --- | --- | --- | --- | --- | --- | --- | --- | --- | --- | --- | --- | --- | --- | --- | --- | --- |
|  | Taking Medicine Total n=68/122, 56% | | Positive  n=33/68, 49% | | No Impact  n=11/68, 16% | | Mixed  n=24/68, 35% | | Self-Management and Other Adherence Targets  Total n=54/122, 44% | | Positive  n=26/54, 48% | | No Impact  n=12/54, 22% | | Mixed  n=16/54, 30% | |
| **Average number of BCTs (M (SD))** | 4.07 (1.94) | | 4.63 (2.01) | | 3.27 (1.95) | | 3.67 (1.66) | | 4.69 (2.70) | | 5.35 (3.11) | | 3.83 (1.99) | | 4.25 (2.29) | |
| **Behavior change technique with examples** | n | % | n | % | n | % | n | % | n | % | n | % | n | % | n | % |
| **Prompts/cues***. Reminder to take medicine, test blood glucose· Push notification or text message sent to user* | 46 | 68 | 25 | 76 | 5 | 45 | 16 | 67 | 39 | 72 | 19 | 73 | 10 | 83 | 10 | 63 |
| **Self-monitoring of outcomes of behavior***. User tracks information about outcomes of behavior (e.g., HbA1c level, weight)*. *Manually entered in app or automatically uploaded via a device (e.g., Bluetooth-enabled glucometer or scale)* | 36 | 53 | 20 | 61 | 4 | 36 | 12 | 50 | 37 | 69 | 19 | 73 | 7 | 58 | 11 | 69 |
| **Feedback on outcomes of behavior***. Graphs of self-monitored outcomes of behavior displayed in app· Communication with care team (user can ask questions, receive feedback on self-monitored data)* | 29 | 43 | 16 | 48 | 4 | 36 | 9 | 38 | 31 | 57 | 18 | 69 | 5 | 42 | 8 | 50 |
| **Self-monitoring of behavior.** *User tracks information about behavior (e.g., taking medicine, exercising, self-testing blood glucose). Manually entered in app or automatically uploaded via a device (e.g., Bluetooth-enabled inhaler, pedometer)* | 33 | 49 | 21 | 64 | 4 | 36 | 8 | 33 | 23 | 43 | 13 | 50 | 4 | 33 | 6 | 38 |
| **Feedback on behavior.** *Graphs of self-monitored outcomes of behavior displayed in app*  *Communication with care team (user can ask questions, receive feedback on self-monitored data)* | 29 | 43 | 19 | 58 | 1 | 9 | 9 | 38 | 17 | 31 | 10 | 38 | 3 | 25 | 4 | 25 |
| **Credible source.**  *Informational content about chronic condition and self-management. Developed and delivered by experts* | 21 | 31 | 13 | 39 | 3 | 27 | 5 | 21 | 20 | 37 | 11 | 42 | 4 | 33 | 5 | 31 |
| **Social support (unspecified)*.*** *Social networking components; elements of cognitive behavioral therapy and motivational interviewing* | 15 | 22 | 7 | 21 | 3 | 27 | 5 | 21 | 12 | 22 | 9 | 35 | 3 | 25 | 0 | 0 |
| **Instruction on how to perform the behavior.** *Skills training (e.g., how to use an inhaler)* | 10 | 15 | 4 | 12 | 3 | 27 | 3 | 13 | 12 | 22 | 8 | 31 | 2 | 17 | 2 | 13 |
| **Goal setting (behavior).** *Set goal for number of times to exercise, log meals, take medicine, etc. in a specified period of time* | 8 | 12 | 6 | 18 | 0 | 0 | 2 | 8 | 11 | 20 | 8 | 31 | 1 | 8 | 2 | 13 |
| **Action planning***. Provide detailed plan for managing medical condition based on data logged in app* | 8 | 12 | 2 | 6 | 2 | 18 | 4 | 17 | 10 | 19 | 5 | 19 | 1 | 8 | 4 | 25 |
| **Information about health consequences.** *Provide information about positive health effects of taking a prescribed daily medication and potential negative consequences of not taking this medicine* | 12 | 18 | 4 | 12 | 1 | 9 | 7 | 29 | 4 | 7 | 2 | 8 | 1 | 8 | 1 | 6 |
| **Conserving mental resources.**  *Provides strategies to reduce the cognitive demands of behavior change (e.g., app contains a user-friendly calculator to recommend bolus insulin dose based on carbohydrate intake)* | 6 | 9 | 3 | 9 | 0 | 0 | 3 | 13 | 7 | 13 | 3 | 12 | 2 | 17 | 2 | 13 |
| **Problem solving.** *Select strategies to address barriers or to facilitate behavior changes (e.g., user reports on barriers to adherence receives text messages with tailored recommendations for overcoming these barriers)* | 7 | 10 | 5 | 15 | 2 | 18 | 0 | 0 | 2 | 4 | 1 | 4 | 0 | 0 | 1 | 6 |
| **Social support (emotional).** *Deliver emotion social support to encourage performance of a health behavior (e.g., user receives text messages with content related to managing emotional distress related to treatment recommendation)* | 2 | 3 | 1 | 3 | 1 | 9 | 0 | 0 | 4 | 7 | 1 | 4 | 0 | 0 | 3 | 19 |
| **Material reward (behavior).**  *Deliver a tangible reward (e.g., money) for completing a specified health behavior (e.g., user earns $1 each day that an electronic pill bottle records medication as "taken")* | 2 | 3 | 1 | 3 | 0 | 0 | 1 | 4 | 4 | 7 | 2 | 8 | 0 | 0 | 2 | 13 |
| **Social support (practical).** *Deliver practical social support to encourage performance of a health behavior (e.g., app contains a care partner/caregiver section with tips for helping to support the user’s adherence or self-management)* | 1 | 1 | 0 | 0 | 0 | 0 | 1 | 4 | 3 | 6 | 1 | 4 | 1 | 8 | 1 | 6 |

*Note.* Percentages do not add up to 100% because studies could use more than one type of behavior change technique.
