# Supplementary figures and images for: Cross-Cutting mHealth Behavior Change Techniques to Support Treatment Adherence and Self-Management of Complex Medical Conditions: Systematic Review
Source: JMIR Mhealth Uhealth. 2024 May 1;12:e49024. doi: 10.2196/49024 (PMC11085043; doi:10.2196/49024)

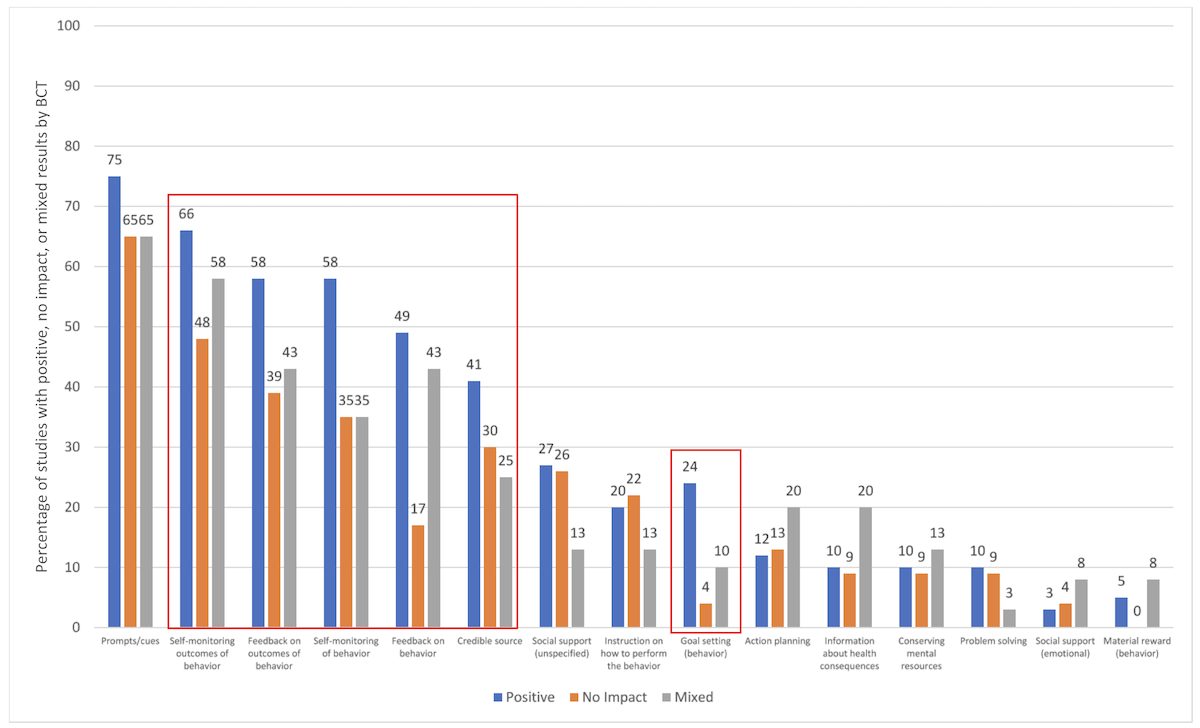

Supplement: Multimedia Appendix 2 [file mhealth-v12-e49024-s002.png]

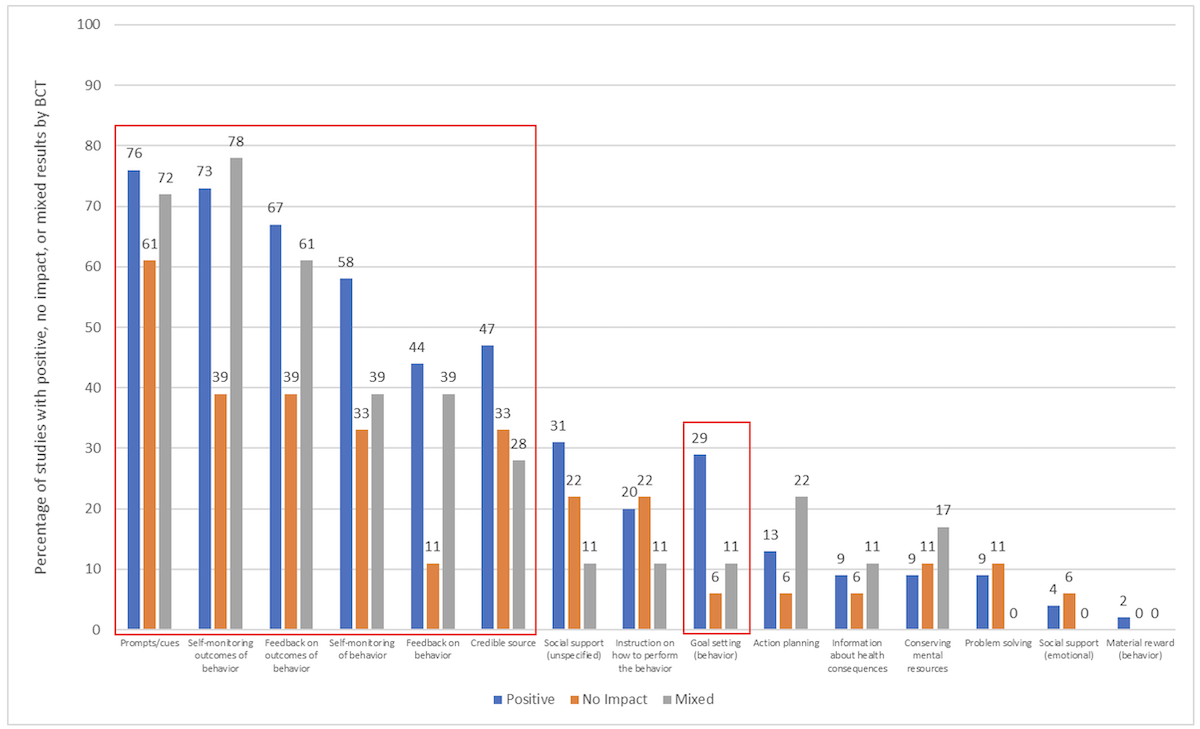

Supplement: Multimedia Appendix 3 [file mhealth-v12-e49024-s003.png]

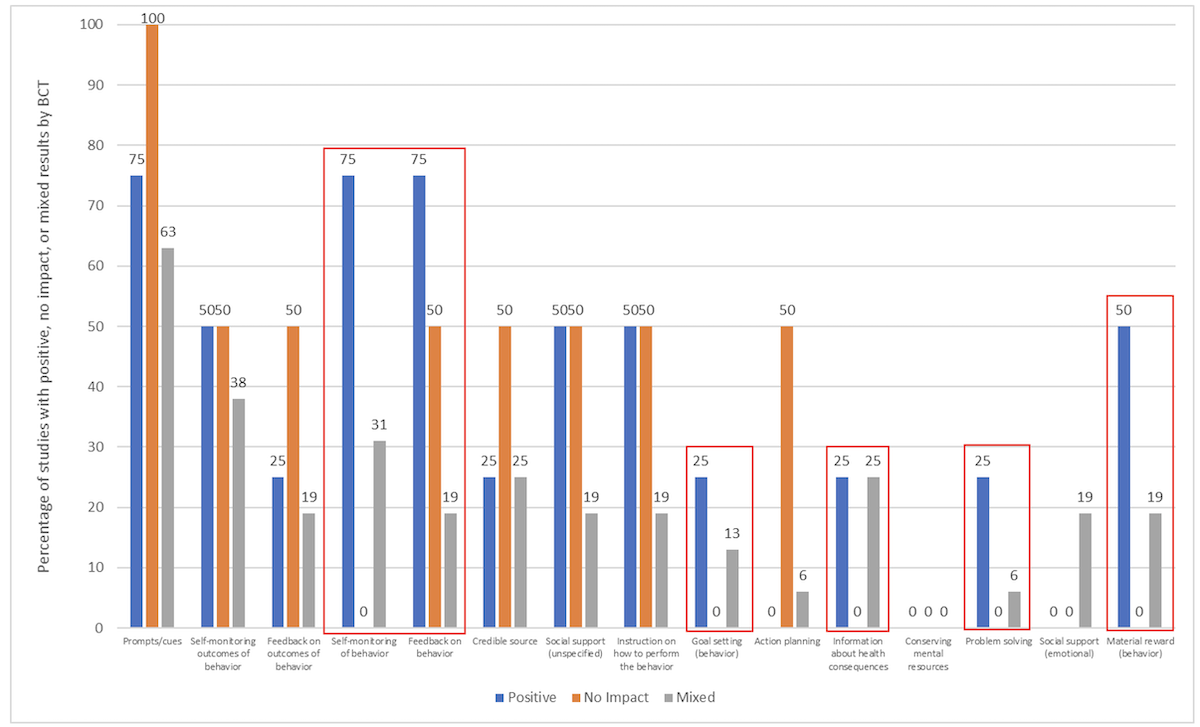

Supplement: Multimedia Appendix 4 [file mhealth-v12-e49024-s004.png]

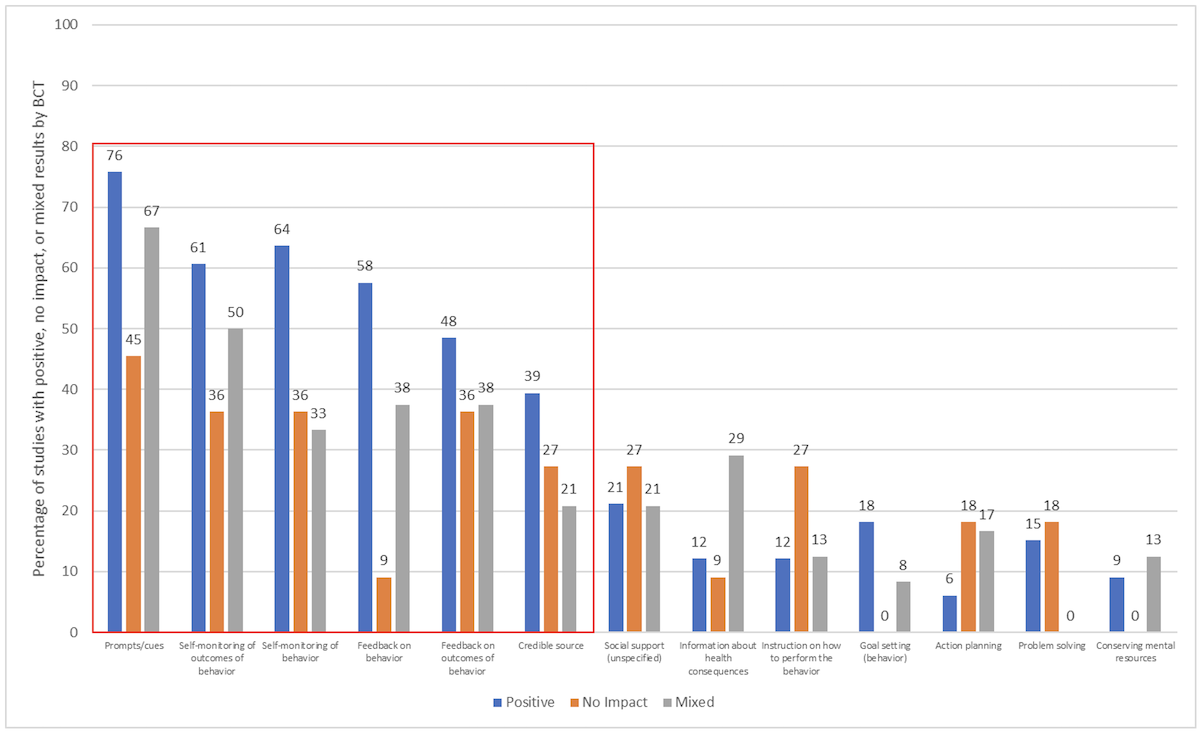

Supplement: Multimedia Appendix 5 [file mhealth-v12-e49024-s005.png]

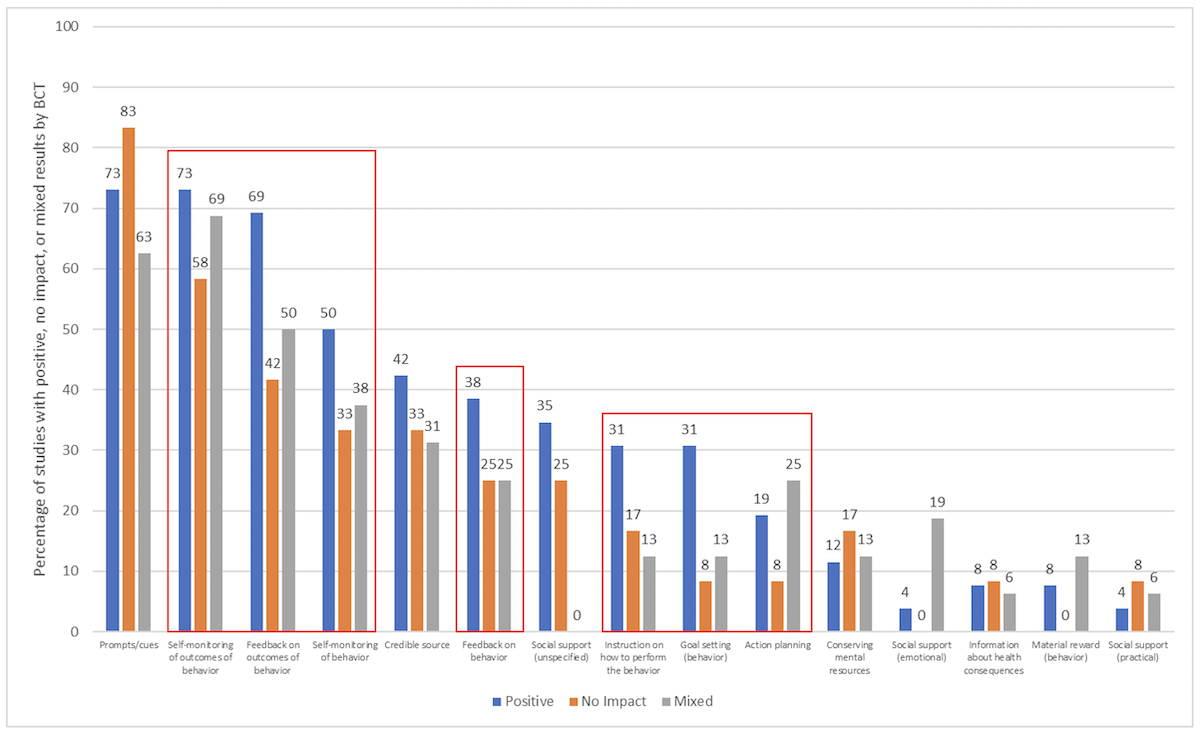

Supplement: Multimedia Appendix 6 [file mhealth-v12-e49024-s006.png]
